# Genomic and biological characterization of a novel bacteriophage X1 infecting *Xanthomonas campestris* pv. *campestris* with biocontrol potential against cabbage black rot

**DOI:** 10.3389/fmicb.2026.1827519

**Published:** 2026-07-07

**Authors:** Qingshan Wu, Ni An, Qing Yu, Xuelian Li, Zheng Fang, Lan Xiang, Qiuping Liu, Leitao Tan, Chuangen Lin, Xiaosheng Zhao, Qingbei Weng

**Affiliations:** 1 School of Life Sciences, Guizhou Normal University, Guiyang, China; 2 School of Biological Sciences and Agriculture, Qiannan Normal University for Nationalities, Duyun, China

**Keywords:** auxiliary metabolic genes, bacteriophage, biocontrol, black rot, genomic characterization, oligotrophic cave, *Xanthomonas campestris*

## Abstract

Bacteriophages have emerged as promising alternatives to pesticides for controlling bacterial pathogens in crops. Black rot, caused by *Xanthomonas campestris* pv. *campestris* (*Xcc*), is one of the most destructive bacterial diseases affecting cruciferous crops worldwide. In this study, a novel lytic bacteriophage, X1, infecting *Xcc* 8004, was isolated from sediment collected in an oligotrophic karst cave. Phage X1 formed clear plaques and exhibited myovirus-like morphology with an icosahedral head and a contractile tail. Biological characterization revealed productive infection of the tested *Xcc* strains but no detectable lytic activity against the non-*Xcc* strains included in the limited host panel. Phage X1 showed rapid adsorption to *Xcc* 8004 (> 99% within 10 min), a latent period of approximately 60 min, and a burst size of approximately 123 plaque-forming units per infected cell. Under laboratory conditions, the phage exhibited stable after incubation at 4–50 °C, pH 5–9, chloroform exposure, and the tested UV-A/UV-C irradiation regimes. Genome sequencing revealed that phage X1 harbors a large linear double-stranded DNA genome of 200,058 bp, encoding 311 predicted open reading frames and 34 tRNA genes. DRAM-v analysis identified 28 candidate auxiliary metabolic genes (AMGs), potentially associated with host metabolic regulation and environmental adaptation. No recognizable antibiotic resistance, virulence, integrase, or lysogeny-associated genes were identified in its genome. Comparative genomic and phylogenetic analyses indicated that X1 is most closely related to *Xanthomonas* phage BUDD within the class *Caudoviricetes*, but represents a distinct species-level genome based on intergenomic similarity. In controlled pot assays, preventive and therapeutic applications of X1 reduced lesion lengths by 88.27 and 73.25%, respectively, compared with *Xcc*-only treatment. Furthermore, preventive application treatment also reduced culturable *Xcc* populations to approximately one-fourth of the level observed after therapeutic treatment. These findings suggest that phage X1 has promising potential for further development as a phage-based biocontrol agent against cabbage black rot.

## 1 Introduction

Plant pathogens infect host plants and cause diseases that substantially reduce crop yields and generate major economic losses worldwide (Savary et al., 2019). Black rot, caused by *Xanthomonas campestris* pv. c*ampestris* (*Xcc*), is one of the most destructive bacterial diseases affecting cruciferous crops globally (Vicente and Holub, 2013). Current management strategies mainly rely on agronomic practices such as crop rotation and the removal of infected plant debris, combined with chemical control measures (Liu et al., 2022; Greer et al., 2023). However, agronomic practices alone often fail to fully prevent disease transmission under field conditions. In addition, the prolonged application of chemical pesticides may promote pathogen resistance and raise environmental and public health concerns, while also contributing to bacterial antimicrobial resistance, thereby reducing the long-term effectiveness of conventional control strategies (Alengebawy et al., 2021; Greer et al., 2023; Ramnarine et al., 2024). Consequently, developing environmentally friendly and sustainable alternatives for black rot management is increasingly important.

Bacteriophage (phage) therapy has recently regained attention as a promising strategy for controlling bacterial plant diseases because phages can specifically infect and lyse their target bacterial hosts (de Leeuw et al., 2020; Stefani et al., 2021). Phages possess several advantages, including high host specificity, self-replication, and excellent environmental compatibility, making them attractive candidates for the biological control of phytopathogenic bacteria (Jain et al., 2023). Several *Xcc*-specific phages have been reported globally, including ɸXF1 from Spain (Vique et al., 2025), MATE2 from Italy (Sabri et al., 2024), and FoX2 and FoX6 from Belgium (Holtappels et al., 2022), all of which demonstrate promising biocontrol potential. However, studies on *Xcc* phages in China remain relatively limited, and few indigenous phages have been characterized for the management of black rot disease. Therefore, the isolation and characterization of novel *Xcc* phages remain necessary for expanding available biocontrol resources.

Extreme environments represent valuable systems for exploring microbial evolution and discovering novel genetic resources (Han et al., 2024). Oligotrophic caves, characterized by long-term environmental stability and extremely low nutrient levels, foster unique microbial communities and serve as promising reservoirs for the discovery of previously unknown phages (Ulbrich et al., 2024). Under nutrient-limited stress, phages often carry auxiliary metabolic genes (AMGs) that are hypothesized to enhance host metabolic capacity and facilitate viral replication (Wang et al., 2024; Liang et al., 2025). Furthermore, environmental pressures in oligotrophic ecosystems may favor phages with relatively large genomes that encode additional functional genes, potentially improving infection efficiency under low host density conditions (Bernard et al., 2025).

In this study, we isolated and characterized a lytic phage, designated as X1, that infects *Xcc* strain 8004. The biological characteristics, morphology, host range, and complete genome of phage X1 were systematically analyzed. Genomic analysis was conducted to identify putative AMGs and explore their potential roles in environmental adaptation and phage-host interactions. Furthermore, the biocontrol efficacy of phage X1 against *Xcc* was evaluated in a controlled pot assay. This study expands the known diversity of *Xcc*-infecting phages and provides a basis for further evaluation of X1 as a candidate biocontrol agent under more realistic agricultural conditions.

## 2 Materials and methods

### 2.1 Sampling, bacterial strain, and culture conditions

Sediment samples for phage isolation were collected in November 2022 from a karst cave in Libo County, Guizhou Province, China, as described previously (Dong et al., 2020). *Xcc* 8004 was stored in 25% (v/v) glycerol at −80 °C in the laboratory. The strain was cultured in King’s B (KB) medium (proteose peptone 20 g, glycerol 10 mL, K_2_HPO_4_ 1.5 g, MgSO_4_ 1.5 g, 1 L distilled H_2_O) at 30 °C overnight, and cells in the exponential phase (2 × 10^7^ colony-forming units per milliliter, CFU/mL) were prepared for phage isolation, enrichment, and plaque assays.

### 2.2 Phage isolation, purification and propagation

Phages were isolated according to the method described previously with minor modifications (Wu et al., 2024). Briefly, 30 g of the sediment was mixed with 270 mL of sterile distilled water and vortexed to achieve a homogeneous mixture. After centrifugation at 6,000 × *g* for 5 min (centrifuge H1650, Xiangyi, China), the supernatant was filtered through a 0.22 μm pore size filter (Merck Millipore, United States), and 9 mL of the filtrate was mixed with 1 mL of *Xcc* 8004 culture. After static adsorption for 10 min at room temperature, the mixture was inoculated into 20 mL of KB medium and then incubated overnight at 30 °C and 160 rpm. The lysate was centrifuged at 6,000 × *g* for 1 min, and the supernatant was filtered through a 0.22 μm filter. One mL of the filtrate and 1 mL of the *Xcc* 8004 culture were mixed, and phage isolation was performed using the double-layer agar plate method. After incubation at 30 °C for 2 days, a single plaque was selected and purified repeatedly until morphologically homogeneous plaques were consistently obtained.

High phage titer stocks were obtained by incubating purified plaques with *Xcc* 8004 culture and plating them on double-agar overlay KB agar plates. The upper layer was harvested and centrifuged (10,000 × *g*, 20 min, 4 °C) followed by filtration using a 0.22 μm filter. The phage particles were precipitated with 10% (w/v) PEG8000 and 0.5 M NaCl overnight at 4 °C, centrifuged at 12,000 × *g* for 20 min. The phage particle precipitate was resuspended in sodium chloride–magnesium sulfate (SM) buffer (50 mM Tris–HCl, pH 7.5, 100 mM NaCl, and 8 mM MgSO_4_·7H_2_O) (Yang et al., 2022). Phage titers were determined by double-layer agar method. The collected phage particles were resuspended in SM buffer and stored at 4 °C. For long-term storage, the phage suspension was maintained in SM buffer supplemented with 25% glycerol at −80 °C (Lee S. Y. et al., 2021).

### 2.3 Transmission electron microscopy

The purified phages (approximately 10^8^ plaque-forming units per milliliter, PFU/mL) were deposited onto carbon-coated copper grids (Daji, Beijing, China), negatively stained with 2% (w/v) potassium phosphotungstate, and visualized by TEM (Hitachi HT7700, Tokyo, Japan) at an accelerating voltage of 100 kV. The phage head and tail dimensions were determined using ImageJ (Wu et al., 2024).

### 2.4 Biological characterization of the phage

#### 2.4.1 Host range

The host range of the phage was preliminarily evaluated using the spot assays, and productive infection of susceptible strains was further assessed by efficiency of plating (EOP) assays. A total of six bacterial strains were tested for host susceptibility. *X. campestris* pv. *campestris* ATCC 33913 and *X. campestris* pv. *badrii* ATCC 11672 were purchased from the Beijing Boyihuike Biotechnology Co., Ltd. (China). *X. campestris* pv. *campestris* XC1 was kindly provided by Professor Kaihuai Li, School of Life Sciences, Guizhou University. *X. oryzae* pv. *oryzae* 2212 was kindly provided by Professor Meiyan Yang, College of Food Science, South China Agricultural University. *Pseudomonas syringae* pv. *tomato* DC3000 was maintained in our laboratory (listed in Supplementary Table S1). Briefly, each strain was cultured in 5 mL of KB medium at 30 °C with shaking to an OD_600_ of 0.5, then centrifuged at 5,000 × *g* for 10 min and resuspended in 5 mL of SM buffer. Then, 0.1 mL of the bacterial suspension was mixed with 4.5 mL of molten soft-agar and poured onto a KB agar plate. After agar solidification, 10 μL of the phage suspension (10^8^ PFU/mL) was spotted onto the plate and incubated for 12 h at 30 °C. The EOP was determined on susceptible bacterial strains by comparing the phage titer obtained on each test strain with that obtained on the reference host *Xcc* 8004. EOP values were classified as: high (EOP ≥ 0.5), moderate (0.1 ≤ EOP < 0.5), and low (0.001 ≤ EOP < 0.1), or undetectable (Khan Mirzaei and Nilsson, 2015).

#### 2.4.2 One step growth curve

A one-step growth curve assay was conducted, following a previously described method with modifications (Fujiki et al., 2021). An exponentially growing *Xcc* 8004 culture (approximately 10^8^ CFU/mL) and the phage suspension were mixed at an multiplicity of infection (MOI) of 0.05 and incubated at 30 °C for 10 min. The mixture was then centrifuged at 10,000 × *g* for 5 min to remove unadsorbed free phages. After being washed with SM buffer, the resulting pellet was resuspended in 30 mL of KB medium and incubated at 30 °C and 180 rpm. The initiation of the infection was designated as t = 0. Four hundred μL of infected cultures were collected every 30 min for 7 h post-infection, followed by filtering through a 0.22 μm filter, and the phage titers were determined. The burst size was calculated as the ratio of the final number of free phage particles to the number of initially infected bacterial cells during the latent period. The experiments were performed with three biological replicates and three technical replicates.

#### 2.4.3 Adsorption rate assay and bacteriostasis test *in vitro*

Overnight cultures of *Xcc* 8004 were collected through centrifugation (5,000 × *g*, 5 min). The pellet was washed and resuspended in KB medium to a final concentration of 10^8^ CFU/mL. The phage suspension was then mixed with the *Xcc* 8004 culture (10^8^ CFU/mL) at an MOI of 0.1, followed by incubation at 30 °C with shaking at 160 rpm. Samples of 300 μL were collected every 5 min for up to 30 min and immediately centrifuged at 10,000 × *g* for 1 min at 4 °C. After filtration through a 0.22 μm filter, the titer of the free phages in the supernatant was measured. The adsorption rate was calculated as: (1 − titer of free phages / initial phage titer) × 100%. For the bacteriostasis test, 100 μL of *Xcc* 8004 (OD_600_ = 0.30) and 100 μL of phage were mixed at various MOIs (100, 10, 1, 0.1, 0.01, 0.001) in a 96-well plate and incubated at 30 °C and 180 rpm. Fresh KB medium without phages was used as the negative control. Absorbance was measured at OD_600_ every 2 h for up to 26 h. All experiments were performed with three biological replicates and three technical replicates.

#### 2.4.4 Thermal, pH, ultraviolet, and chloroform stability

The thermal stability was determined by incubating 10^8^ PFU/mL of phage in a water bath at various temperatures (4, 20, 30, 40, 50, 60 and 70 °C) for 1 h, respectively. The phage titer from collected samples was determined by a double-layer method. To assess pH stability, 100 μL of phage suspension (10^8^ PFU/mL) was mixed with 900 μL of SM buffer at specific pH values ranging from 3 to 12 (adjusted using 1 mol/L HCl or 1 mol/L NaOH), followed by incubation at 30 °C for 1 h and then phage titers were measured. To evaluate the effects of UV irradiation at 365 nm on the phage, a phage suspension (10^8^ PFU/mL) was placed 20 cm from ultraviolet lamps (lamp length 41 cm, 36 W, Philips, Holland) for 1, 2, 3, 4, 5, 6, 7, 8, 9, and 10 h. Additionally, to assess the effects of UV irradiation at 254 nm, the phage suspension (10^8^ PFU/mL) was placed 20 cm from ultraviolet lamps (lamp length 50 cm, 36 W, Philips, Holland) for 0, 20, 40, 60, 80, 100, and 120 min. To determine the sensitivity of the phage to chloroform, the phages (10^8^ PFU/mL) were incubated with 25% chloroform on a shaker for 1 h. The control group lacked chloroform. The phage titers were determined by the double-layer agar method. All experiments were carried out in triplicate.

### 2.5 Extraction of phage genome and sequencing

The genomic DNA of the phage was extracted from purified phage lysates (10^8^ PFU/mL) using the viral genome extraction kit (Takara, Japan) following the manufacturer’s instructions. The extracted DNA was sent to Shanghai Personal Biotechnology Co., Ltd. (China) for sequencing and assembly. Briefly, a DNA library was constructed using the TruSeqTM DNA Sample Prep Kit (Illumina, San Diego, CA, United States) and sequenced in paired-end mode using the Illumina NovaSeq platform. The obtained FastQ raw reads were trimmed of adaptors and low-quality bases and short reads were filtered using AdapterRemoval v2.1.3 (Schubert et al., 2016). Clean reads were then *de novo* assembled using A5-MiSeq v20160825 and SPAdes v3.12.0 (Bankevich et al., 2012; Coil et al., 2015). Subsequently, the contigs were further assembled into complete genome sequence for the phage using Blast v2.12.0, MUMmer v3.1 and Pilon v1.18 (Altschul et al., 1990; Kurtz et al., 2004; Walker et al., 2014).

### 2.6 Genome analysis

The phage genome was submitted for sequence similarity searches using BLAST, the alignments were further evaluated by query coverage, identity and similarity. Open reading frames (ORFs) were predicted using the GeneMark webserver^1^ and RAST annotation engine.^2^ Each predicted ORF was annotated by performing a search against the NCBI non-redundant (nr) protein database and conserved domain database using BLASTp.^3^ Antibiotic resistance genes and virulence factors were predicted by alignment with the Comprehensive Antibiotic Resistance Database (CARD, https://card.mcmaster.ca/analyze/rgi) and Virulence Factor Database (VFDB, http://www.mgc.ac.cn/VFs/) (Liu et al., 2019; Alcock et al., 2023). The availability of tRNA was analyzed by the tRNAScan-SE webserver.^4^ The genome was constructed and visualized using the Proksee Server.^5^ The protein-coding sequences of phage X1 were annotated using DRAM-v (Shaffer et al., 2020) with default parameters to predict putative AMGs and obtain KEGG Orthology (KO) identifiers. Genes with an auxiliary score below 4, a metabolic annotation, and no viral structural or accessory tags were defined as considered candidate AMGs. The acquired KO entries were further mapped to metabolic pathways via KEGG mapper for pathway reconstruction and functional analysis of phage AMGs (Pratama et al., 2026).

### 2.7 Phylogenetic analysis and comparative genomic analysis

To investigate the evolutionary relationship between phage X1 and other phages, all reference phage sequences exhibiting the highest similarity to the complete genome sequence of phage X1, as identified through NCBI BLASTn analysis, were selected for phylogenetic analysis in this study. The phylogenetic tree was constructed by the Maximum Likelihood method using MEGA 12.1.2 (Kumar et al., 2016) with the JTT matrix-based model and 1,000 bootstrap replicates based on the amino acid sequences of the terminal large subunit. A proteome phylogenetic tree was constructed using the Viral Proteomic Tree server (ViPTree, https://www.genome.jp/viptree/) (Nishimura et al., 2017). The genome-wide average nucleotide identity (ANI) between phage X1 and its closely related phages was calculated and plotted by the Virus Intergenomic Distance Calculator (VIRIDIC) (Moraru et al., 2020). Comparative genomic analysis of the whole genome sequences of phage X1 and its closest relative was performed at the gene level using Easyfig v3.4 (Sullivan et al., 2011).

### 2.8 *In planta* antagonistic effects of X1 against *Xcc* 8004

To further investigate the biocontrol efficacy of phage X1 against *Xcc* 8004, a foliar spraying method was employed (Skliros et al., 2023). *Brassica oleracea* var. *capitata* “Jingfeng No. 1”, which is susceptible to *Xcc* 8004, was used. The cabbage seedlings were transplanted into pots (10 cm in diameter × 10 cm in height) and grown for 5 weeks in a growth chamber under controlled conditions (25 °C, 75% relative humidity, 16:8 h light–dark cycle). *Xcc* 8004 was cultured in KB medium supplemented with 50 μg/mL rifampicin for 24 h at 30 °C and 180 rpm, followed by resuspension in magnesium chloride solution to achieve an optical density of 0.2 at 600 nm (approximately 10^8^ CFU/mL). Phage X1 was propagated with *Xcc* 8004 as the host strain, and the resulting lysate was filtered through a 0.22 μm filter membrane to obtain the phage preparation.

In the potted experiment, five treatment groups were established: (1) the control group (CK), treated with 10 mM magnesium chloride solution; (2) the *Xcc* treatment group (*Xcc*), treated with 1 mL of *Xcc* 8004 culture (OD_600_ = 0.2) as a positive control; (3) the phage X1 treatment group (X1), treated with 1 mL of phage X1 preparation (5.7 × 10^8^ PFU/mL); (4) the *Xcc* + X1 treatment group (*Xcc +* X1), inoculated with *Xcc* 8004 (OD_600_ = 0.2) for 24 h prior to treatment with phage X1 (5.7 × 10^8^ PFU/mL); and (5) the X1 + *Xcc* treatment group (X1 + *Xcc*), treated with phage X1 for 24 h before inoculation with *Xcc* 8004. Each group contained six cabbage plants serving as biological replicates (*n* = 6). For each plant, four fully expanded leaves were wounded by cutting the leaf tips, and the wounded sites were then sprayed with the *Xcc* 8004 suspension or biocontrol agents (Holtappels et al., 2022). All plants were cultured in a biochemical incubator at 30 °C for 14 days under 90% relative humidity. After inoculation, disease symptoms were observed, and lesion length was measured using ImageJ software. The average lesion length of four leaves per plant was calculated as the representative value for each biological replicate. At 14 days post-inoculation (dpi), the same 24 leaves (four leaves per plant × six plants) per group were collected to assess *Xcc* 8004 concentration. The four leaves collected from a single plant were pooled as one sample. Leaf samples were surface-sterilized with 70% ethanol for 1 min, rinsed with ddH_2_O for 30 s, and the diseased tissues were homogenized. From each homogenate, 0.5 g of leaf tissue was mixed with 1 mL sterile water and further homogenized. The leaf homogenate was serially 10-fold diluted, and three technical replicates of each dilution were inoculated onto KB plates containing 50 μg/mL rifampicin. The cultures were incubated at 30 °C for 48 h. Since *Xcc* 8004 carries a stable rifampicin-resistance marker, rifampicin-containing plates were used to selectively recover the rifampicin-resistant *Xcc* 8004 strain and reduce background growth from plant-associated bacteria. Subsequently, rifampicin-resistant colonies were counted to determine the number of colony-forming units. Statistical analyses were performed using the six biological replicate means per treatment group.

### 2.9 Statistical analysis

All experiments were performed with three biological replicates. One- or two-way analysis of variance and the resulting plots were performed using GraphPad Prism. The results are expressed as the mean ± SD. A *p* < 0.05 was considered statistically significant.

## 3 Results

### 3.1 Isolation and morphology of phage X1

**Figure 1:**
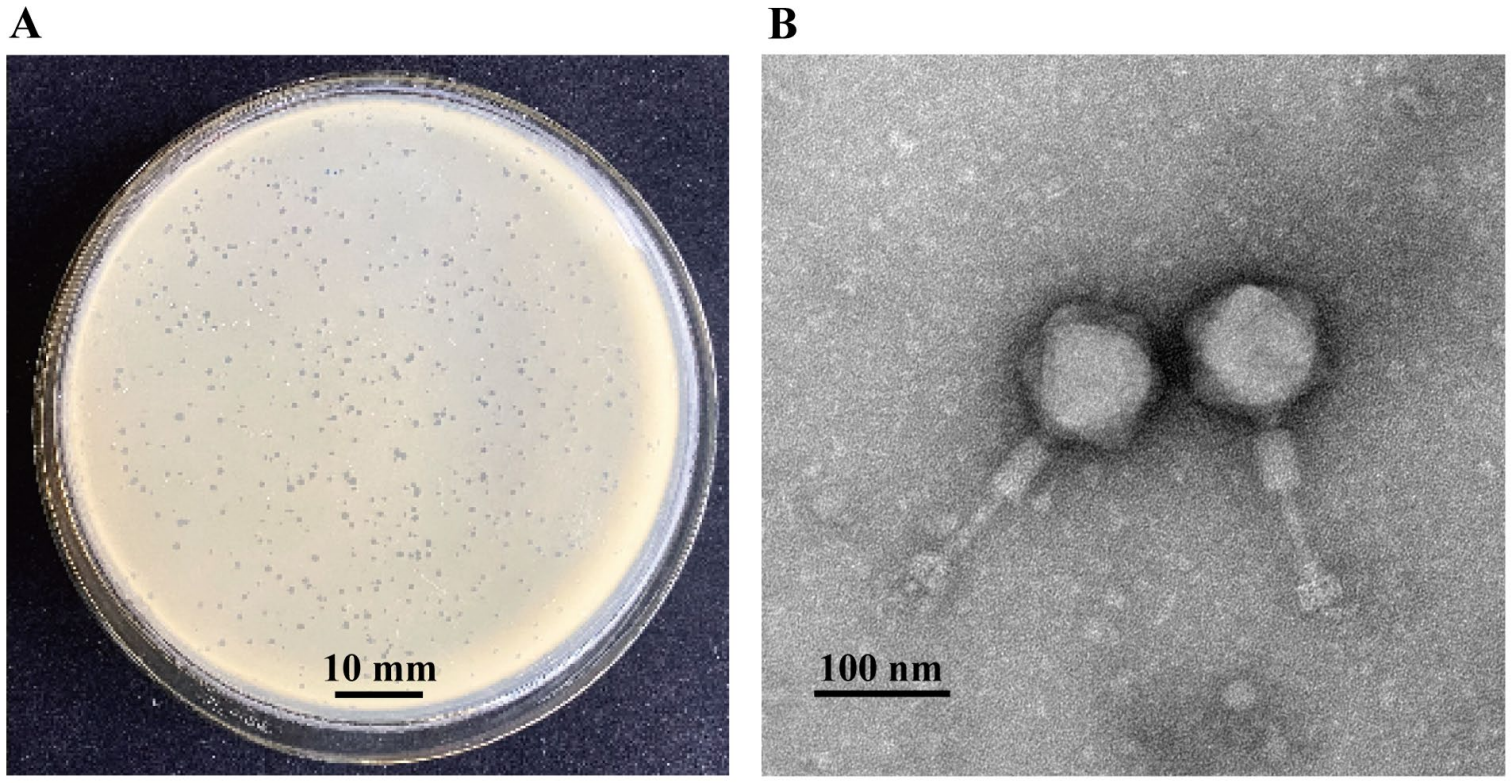
Morphology of phage X1. **(A)** Plaque morphology. Purified phage X1 was mixed with the host *Xcc* 8004 culture and plated on double-layer KB agar plates. The plates were incubated at 30 °C for 2 days. Clear, circular plaques representing lytic zones formed on the bacterial lawn. Scale bar = 10 mm. **(B)** Morphology of phage X1 as revealed by TEM. Purified phage X1 particles (approximately 10^8^ PFU/mL) were deposited onto carbon-coated copper grids, negatively stained with 2% (w/v) potassium phosphotungstate, and observed under a TEM (Hitachi HT7700) at an accelerating voltage of 100 kV. The image shows the phage morphology comprising an icosahedral head and a contractile tail. Head and tail dimensions were measured using ImageJ software. Scale bar = 100 nm.

A lytic phage infecting *Xcc* 8004, designated X1, was isolated from cave sediments collected in Guizhou, China. Phage X1 produced small, clear, and circular plaques approximately 2 mm in diameter on *Xcc* 8004 lawns after incubation at 30 °C (Figure 1A). TEM revealed that phage X1 possessed a myovirus-like morphology, with an icosahedral head (99 ± 4 nm in diameter) and a long contractile tail (143 ± 1 nm in length) (Figure 1B).

### 3.2 Host range, one step growth curve and adsorption rate assays

Host range analysis using spot assays suggested that phage X1 exhibited lytic activity against three *Xcc* strains (XC1, ATCC 33913, and 8004), but not against the non-*Xcc* strains tested, including *X. campestris* pv. *badrii* ATCC 11672, *X. oryzae* pv. *oryzae* 2212, and *P. syringae* pv. *tomato* DC3000. Productive infection was supported by EOP assays on susceptible strains, XC1 and ATCC 33913 displayed moderate sensitivity, with EOP values ranging from 0.1 to 0.5 (Supplementary Table S1).

**Figure 2:**
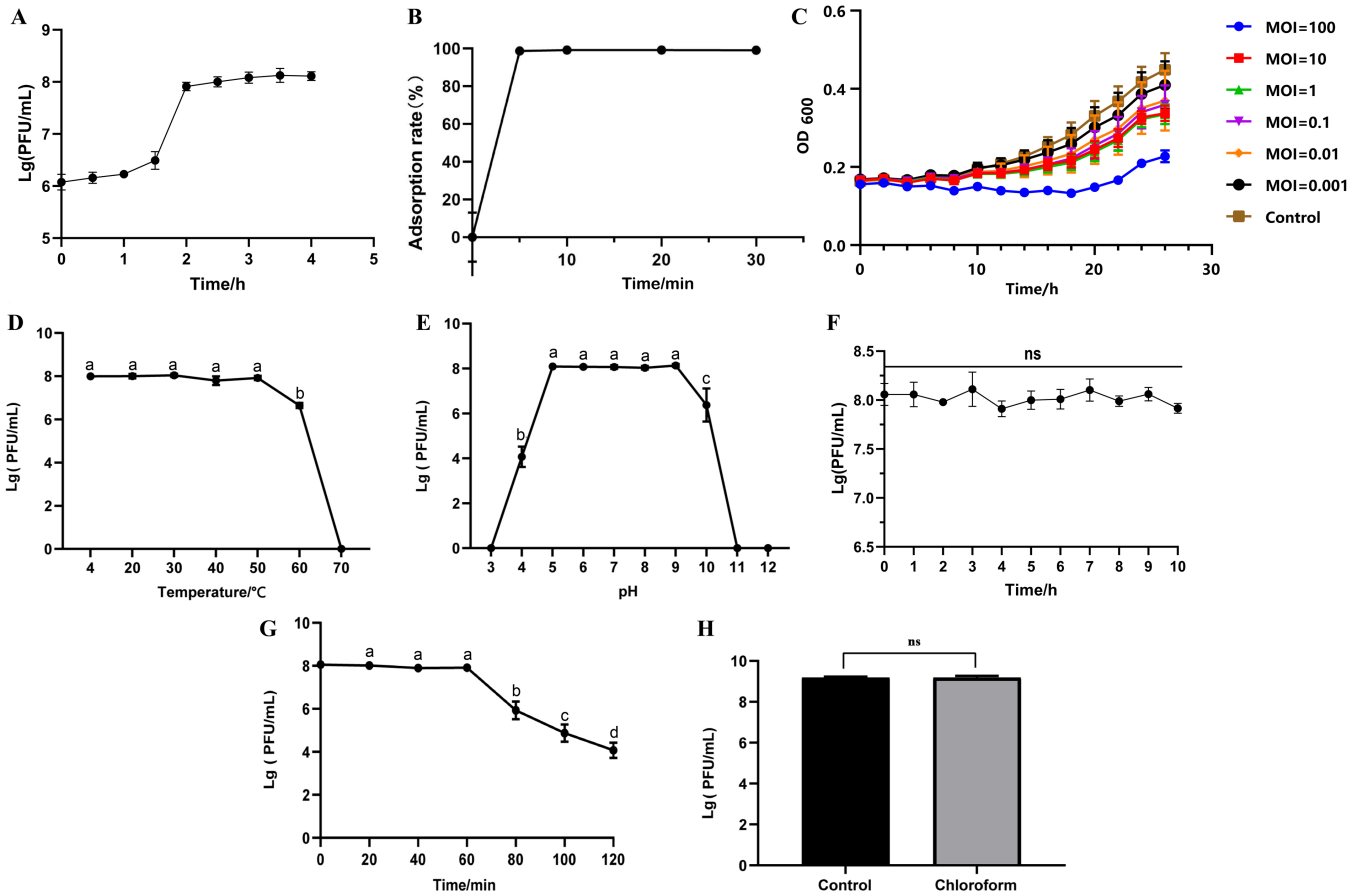
Biological characteristics of phage X1. **(A)** One-step growth curve of phage X1. Phage X1 was mixed with the host *Xcc* 8004 at MOI of 0.05. After adsorption (10 min at 30 °C) and removal of unadsorbed phages, infected cells were resuspended in KB medium and incubated at 30 °C with shaking (180 rpm). Samples were collected every 30 min for 5 h post-infection, and phage titers were determined by plaque assay. The logarithmic phage concentration (log_10_ PFU/mL) was plotted against time (hours post-infection). **(B)** Adsorption rate of phage X1. Phage X1 and *Xcc* 8004 (final concentration of 10^8^ PFU/mL and 10^8^ CFU/mL, respectively) were mixed at an MOI of 0.1 and incubated at 30 °C with shaking (160 rpm). At indicated time points (0, 5, 10, 15, 20, 25, and 30 min), samples were collected and immediately centrifuged; free phages in the supernatant were titrated. Adsorption rate (%) = (1 – titer of free phages / initial phage titer) × 100%. The adsorption rate (%) was plotted against time (minutes post-infection). **(C)** *In vitro* bacteriolytic activity of phage X1 against the host *Xcc* 8004. *Xcc* 8004 culture (OD_600_ = 0.30) was mixed with phage X1 at different MOIs (100, 10, 1, 0.1, 0.01, 0.001) in a 96-well plate and incubated at 30 °C with shaking (180 rpm). Bacterial growth was monitored by measuring OD_600_ every 2 h for up to 26 h. KB medium without phage served as the control. The OD₆₀₀ was plotted against time (hours post-infection). **(D)** Temperature stability. Phage X1 suspension (10^8^ PFU/mL) was incubated in a water bath at different temperatures (4, 20, 30, 40, 50, 60, and 70 °C) for 1 h. Residual phage titers were determined by double-layer agar method. The logarithmic phage titer (log_10_ PFU/mL) was plotted against temperature (°C). **(E)** pH stability. Phage X1 (10^8^ PFU/mL) was mixed with SM buffer adjusted to pH values from 3 to 12 (increments of 1) at a 1:9 ratio and incubated at 30 °C for 1 h. Residual phage titers were then measured. The logarithmic phage titer (log_10_ PFU/mL) was plotted against pH. **(F)** Ultraviolet (UV-A) stability. Phage X1 suspension (10^8^ PFU/mL) was placed 20 cm below a UV lamp (365 nm, 36 W) and exposed for 1, 2, 3, 4, 5, 6, 7, 8, 9 and 10 h. Residual phage titers were determined. The logarithmic phage titer (log_10_ PFU/mL) was plotted against UV exposure time (minutes). **(G)** Ultraviolet (UV-C) stability. Phage X1 suspension (10^8^ PFU/mL) was placed 20 cm below a UV lamp (254 nm, 36 W) and exposed for 0, 20, 40, 60, 80, 100 and 120 min. Residual phage titers were determined. The logarithmic phage titer (log_10_ PFU/mL) was plotted against UV exposure time (minutes). **(H)** Chloroform stability. Phage X1 (10^8^ PFU/mL) was incubated with 25% (v/v) chloroform on a shaker for 1 h. The control group received no chloroform. Residual phage titers were measured. The bar chart shows the titer (PFU/mL) for the control and chloroform-treated groups. All data are presented as the mean of triplicate experiments, and the error bars indicate the standard deviation. Statistical significance was determined using one-way or two-way ANOVA and followed by Dunnett’s *post hoc* test. Different letters (a, b, c, d) indicate statistically significant differences among treatments at *p* < 0.05; ns, no significant difference (*p* > 0.05).

The one-step growth curve revealed a latent period of approximately 60 min followed by a lysis period of 90 min, with an average burst size of approximately 123 PFU per infected cell (Figure 2A). Phage X1 rapidly adsorbed to *Xcc* 8004, reaching an adsorption efficiency of 98.78% within 5 min and further increasing to 99.22% after 10 min (Figure 2B), indicating highly efficient adsorption to the host strain.

The inhibitory effect of phage X1 on *Xcc* 8004 was further evaluated *in vitro* at different MOIs (Figure 2C). Bacterial growth inhibition was MOI-dependent, and at an MOI of 100, phage X1 exerted a stable bacteriostatic effect on the host bacterial population.

### 3.3 Stability and *in vitro* bacteriostatic activity of phage X1

The environmental stability of phage X1 was evaluated, under different temperatures, pH conditions, UV exposure times and chloroform treatment. Phage X1 remained stable at temperatures ranging from 4 °C to 50 °C (*p* > 0.05), maintaining a titer of 7.21 × 10^8^ PFU/mL. As the temperature increased, the titer gradually decreased to 4.30 × 10^6^ PFU/mL at 60 °C, and the phage was completely inactivated at 70 °C (Figure 2D), indicating good thermal stability. Phage X1 also showed tolerance to both acidic and alkaline environments. After 1 h of incubation, the titer remained approximately 10^8^ PFU/mL at pH 5 and pH 9 (*p* > 0.05), but declined significantly at pH 10. Complete inactivation occurred at pH ≤ 3 or ≥11 (Figure 2E). Phage X1 retained infectivity (*p* > 0.05, Figure 2F) after the tested 365-nm UV-A exposure regime, while under 254 nm irradiation, the phage titer gradually decreased with increasing exposure time but remained detectable at 4.39 × 10^4^ PFU/mL after 120 min (Figure 2G). Treatment with 25% chloroform had no significant effect on phage activity compared with the control group (*p* > 0.05, Figure 2H).

### 3.4 General genome characteristics and functional ORF annotation of phage X1

Whole-genome sequencing of phage X1 generated 4,305,064 filtered reads, which were assembled into a complete genome. The genome possesses a linear double-stranded DNA molecule of 200,058 bp with a G + C content of 50.21%. The sequence has been deposited in GenBank under the accession number OQ427096.1.

Genome annotation identified 311 ORFs. Among these, 291 ORFs (93.57%) initiate with ATG, 12 ORFs (3.86%) with GTG, and 8 ORFs (2.57%) with TTG (Supplementary Table S3). Of the total ORFs, 281 are located on the positive strand and 30 on the negative strand. The coding sequences span 180,462 bp with an average length of 580.26 bp, representing 90.20% of the genome. No recognizable virulence, antimicrobial resistance, integrase, or lysogeny-associated genes were identified with the tools used.

**Figure 3:**
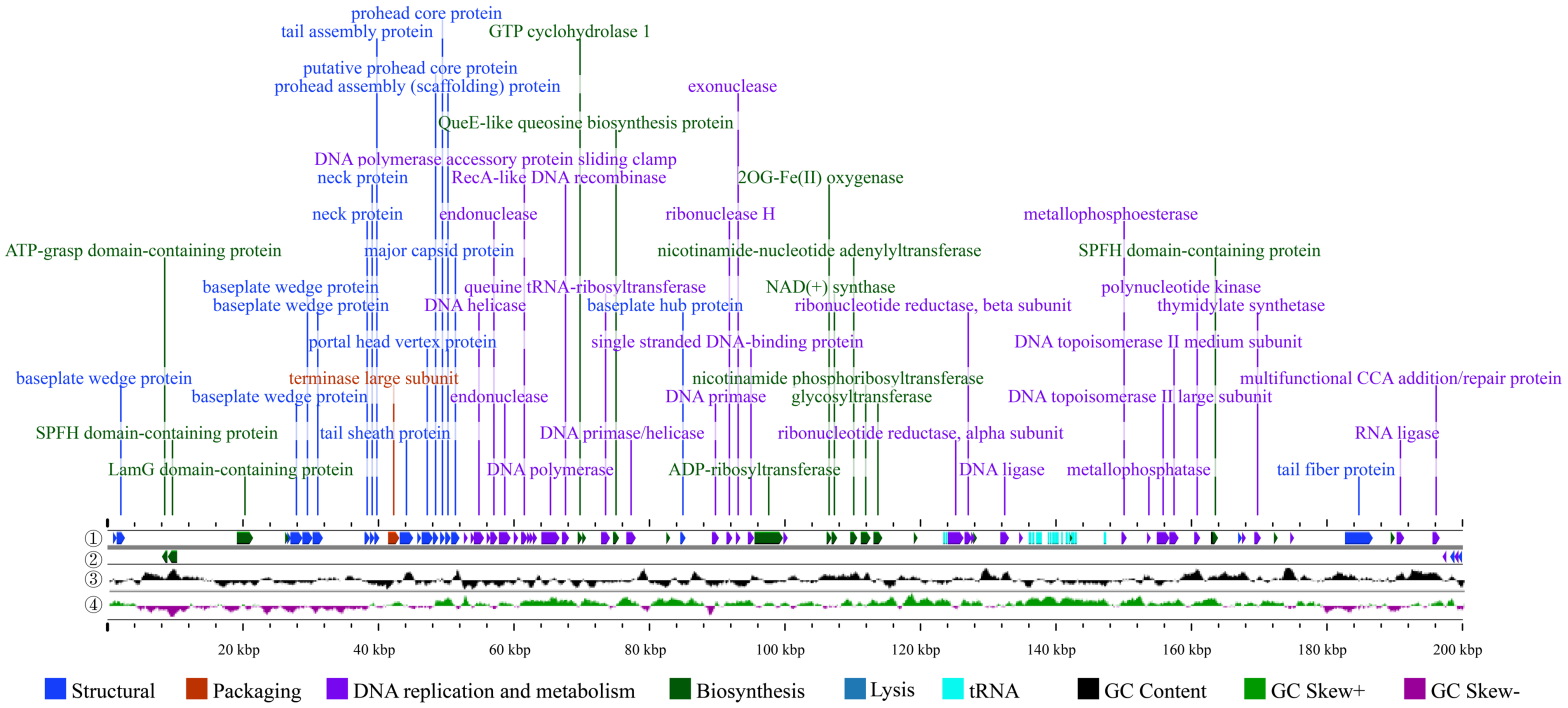
Genome map of phage X1. The complete genome of phage X1 was sequenced using the Illumina NovaSeq platform (paired-end mode). Raw reads were trimmed and assembled using A5-MiSeq and SPAdes, then further assembled into a complete circular genome using BLAST, MUMmer, and Pilon. ORFs were predicted using GeneMark and RAST, and annotated by BLASTp against the NCBI nr database and conserved domain database. tRNA genes were predicted using tRNAScan-SE. The genome map was constructed and visualized using Proksee. Rings ① and ② indicate the ORFs on the positive and negative strands. The different colors represent different gene functions. Ring ③ indicates the GC content, and ring ④ represents the GC Skew value.

Among the 311 predicted ORFs, 78 were assigned putative functions, 216 were annotated as hypothetical proteins, and 17 showed no significant similarity to any known proteins in BLASTp analysis. Based on functional annotation, the genome was organized into five modules (Figure 3): structural proteins (19 ORFs), DNA replication and modification (36 ORFs), host metabolism-related genes (19 ORFs), DNA packaging (2 ORFs), and lysis (2 ORFs) (Supplementary Tables S2, S3).

Within the structural module, six ORFs (ORF1, ORF43, ORF47, ORF49, ORF247, and ORF281) encode tail-associated proteins, while ORF41 and ORF42 encode neck proteins. Six ORFs (ORF2, ORF35, ORF36, ORF37, ORF103 and ORF309) encode baseplate assembly proteins, and five ORFs (ORF 50, 51, 53, 54, and 311) encode head proteins. Tail fiber and baseplate proteins typically function as receptor-binding proteins involved in host recognition.

The DNA replication and modification module contains genes encoding DNA polymerase, deoxyuridine triphosphatase, RecA-family DNA recombinases, RNaseH endonuclease, DNA ligase, RNA ligase, DNA topoisomerase II, single-stranded DNA-binding protein, ribonucleotide reductase subunits *α* and *β*, nucleoside 2′-deoxyribose transferase, and other replication-associated enzymes.

The putative host metabolism-related genes module includes genes encoding an ATP-grasp domain protein, SPFH (stomatin, prohibitin, flotillin, and HflK/C) domain proteins, guanosine triphosphate cyclohydrolase I, adenosine phosphate sulfate reductase family proteins, adenylyltransferase family protein, ADP-ribotransferase, oxoglutarate oxygenase, glutaredoxin, and the GroES molecular chaperone.

The lysis module includes a transmembrane protein encoded by ORF234 and a cell wall hydrolase encoded by ORF288.

Additionally, 34 tRNA genes corresponding to 18 amino acids were identified in the genome. Multiple tRNAs were identified for glutamine, glutamic acid, glycine, isoleucine, leucine, lysine, methionine, phenylalanine, proline, threonine, tyrosine, and valine (Supplementary Table S4).

### 3.5 AMGs of phage X1

A total of 28 putative AMGs were predicted in the genome of phage X1 by DRAM-v (Supplementary Table S5). These genes are primarily involved in amino acid metabolism and biosynthesis (5 genes), cofactor synthesis (9 genes), substance transport (6 genes), sulfur metabolism (2 genes), and cell wall and exopolysaccharide biosynthesis (6 genes). Genes associated with amino acid metabolism and biosynthesis include *TDH*, *serK*, *argG*, *asnB*, and *GTK*. Cofactor synthesis genes comprise *FLAD1*, *ribF*, *pncB*, *nadM*, *nadD*, *nadE*, *NAMPT*, *coaE*, and *thiF*. Transport-related genes include *modF*, *tupC*, *nikE*, *wtpC*, *btuD*, and *ecfA1*. Sulfur metabolism genes include *cysD* and *cysH*, while genes contributing to cell wall and exopolysaccharide biosynthesis include *gumH*, *wcaC*, *lpxH*, *tagE*, *mgs*, and *tarM*.

### 3.6 Phylogenetic analysis of phage X1

BLASTn analysis of the complete genome showed that phage X1 shared the highest similarity with *Xanthomonas* phage BUDD (GenBank: ON758385.1), with 86.99% nucleotide identity and 95% genome coverage. Additionally, phage X1 showed similarity to several *Stenotrophomonas* phages, with nucleotide identities ranging from 72.48 to 74.96%.

**Figure 4:**
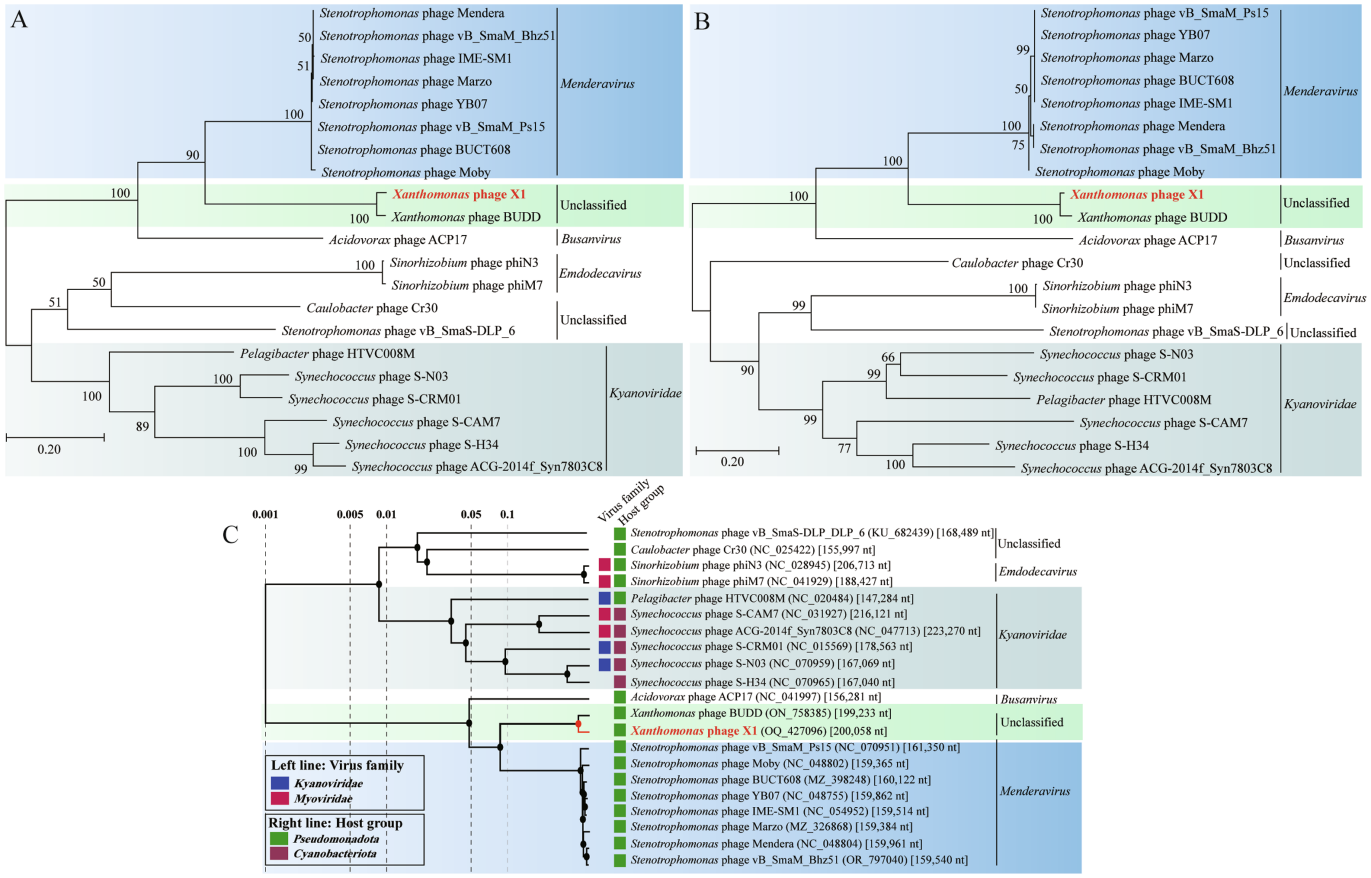
Phylogenetic analysis of phage X1. Phylogenetic trees were constructed based on the terminase large subunits **(A)** and DNA polymerase **(B)** for phage X1 using the Maximum Likelihood method in MEGA 12.1.2. The bootstrap values were based on 1,000 replicates and are shown near their branches. Bootstrap values are shown near their branches. Branches with a bootstrap support lower than 50% were deleted. The reference sequences were retrieved from the NCBI database. **(C)** Proteome phylogenetic analysis of phage X1 and the closely related phages performed using VipTree. Branch lengths are shown on a logarithmic scale from the root of the tree. The inner nodes of the tree are shown as filled circles, each links to a genomic alignment of the sequences included in its subtree. The left panel indicated the virus family, and the right panel indicated the host group. The red text represents *Xanthomonas* phage X1 isolated in this study.

Phylogenetic trees constructed using the terminase large subunit (Figure 4A), DNA polymerase I (Figure 4B), and whole-proteome analysis (Figure 4C) exhibited consistent topologies. Phage X1 clustered with the unclassified *Xanthomonas* phage BUDD and further grouped with several members of the genus *Menderavirus* within the class *Caudoviricetes*, including eight *Stenotrophomonas* phages: vB_SmaM_Ps15 (NC_070951), Moby (NC_048802), BUCT608 (MZ_398248), YB07 (NC_048755), IME-SM1 (NC_054952), Marzo (MZ_326868), Mendera (NC_048804), and vB_SmaM_Bhz51 (OR_797040).

**Figure 5:**
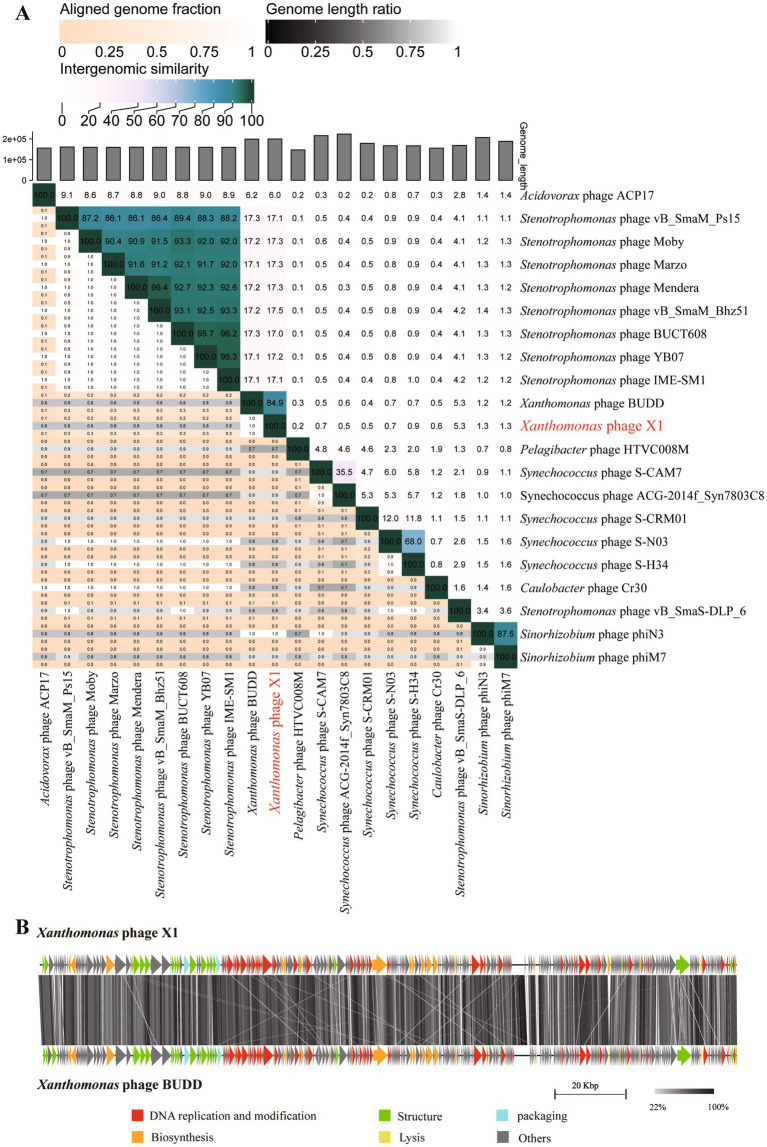
Genomic similarity of phage X1. **(A)** Intergenomic similarity between X1 and related phages, calculated using VIRIDIC. The right half of this heatmap shows the similarity values between phage genomes. The left half of this heatmap shows the aligned genome fraction and genome length ratio. The red highlight indicates phage X1 from this study. **(B)** Pairwise comparisons of the whole genomes of *Xanthomonas* phage X1 and phage BUDD was visualized using Easyfig. Arrows indicate encoded proteins and the grey line connecting the two genomes represent the similarity between the two, with darker shades of grey representing higher similarity.

VIRIDIC analysis revealed that intergenomic similarity between phage X1 and related phages, as determined by VIRIDIC, ranged from 0.2 to 84.9%, with the highest value observed between phage X1 and phage BUDD (Figure 5A). This value is below the 95% species demarcation threshold defined by the International Committee on Taxonomy of Viruses Bacterial Virus Subcommittee (Turner et al., 2021), indicating that phage X1 represents a novel phage species within the class *Caudoviricetes*.

Comparative genomic analysis further demonstrated strong collinearity between phage X1 and phage BUDD (Figure 5B). Among the proteins encoded by phage X1, 290 (93.25%) shared sequence similarity with those encoded by phage BUDD, with sequence identities ranging from 22 to 100%, although several genes exhibited positional rearrangements.

### 3.7 Evaluation of plant protective activity of phage X1 against *Xcc* 8004 *in planta*

**Figure 6:**
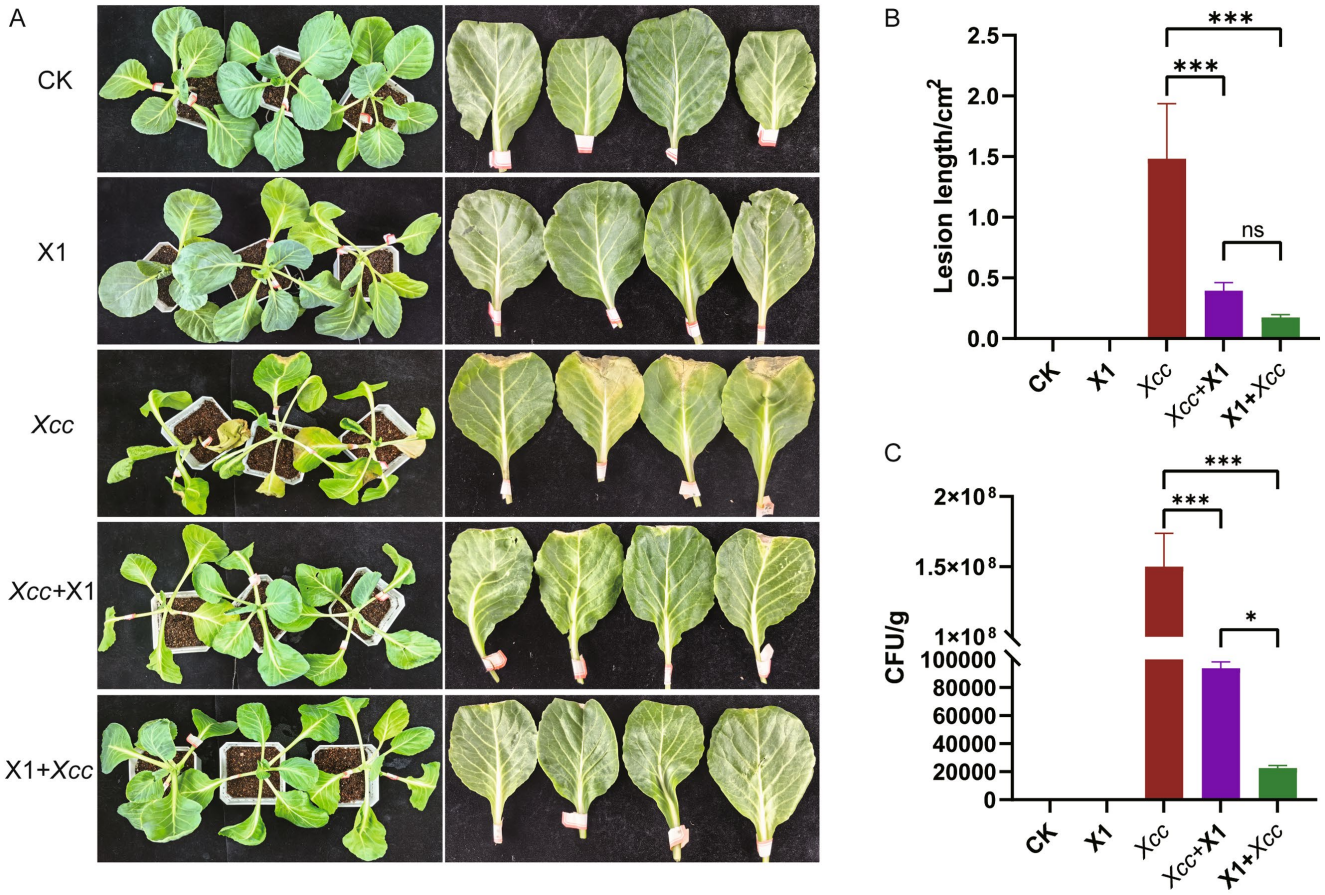
Biocontrol evaluation of phage X1 against *Xcc* 8004 *in planta*. **(A)** Cabbage seedling assay to determine the inhibitory effect of the phage on the pathogenicity of *Xcc* 8004. Four fully expanded leaves per plant were wounded by cutting the leaf tips, and the wounded sites were then sprayed with the *Xcc* 8004 suspension or biocontrol agents. Treatments were designated as follows: the blank control (CK), phage treatments alone (X1), the positive control (*Xcc*), and the co-treatment groups of phage (*Xcc*+X1, X1 + X*cc*). The *Xcc* + X1 treatment group was treated with *Xcc* 8004 for 24 h prior to treatment with phage X1 (therapeutic treatment). The X1 + *Xcc* treatment group was treated with phage X1 for 24 h before being treated with *Xcc* 8004 (preventive treatment). Each treatment used six cabbage plants as biological replicates (The figure only shows 3 complete plants for each treatment, and the leaves only display 4 leaves of one plant). Leaf symptoms were observed on 3, 7, 10 and 14 dpi. **(B)** Measurement of the lesion length on the leaves using ImageJ software. At 14 dpi, the average lesion length of four leaves per plant was calculated as the representative value for each biological replicate. **(C)** After 14 days of treatment, the leaves of the plants were collected to determine the bacterial load of *Xcc* 8004. For each plant, the four leaves were pooled as one biological replicate. Samples were surface-sterilized, homogenized, and 0.5 g of homogenate was mixed with 1 mL sterile water. Ten-fold serial dilutions were plated (three aliquots per dilution) on KB agar with 50 μg/mL rifampicin to selectively recover *Xcc* 8004. After 48 h at 30 °C, rifampicin-resistant colonies were counted. The data are shown as the mean ± SD. Statistically significant differences among the treatments are indicated by * (*p* < 0.05) and *** (*p* < 0.001) for the bar plot, ns indicates no significance.

The biocontrol efficacy of phage X1 against bacterial black rot caused by *Xcc* 8004 was evaluated using a potted plant leaf-cutting and spraying method. Disease symptoms were observed at 3, 7, 10, and 14 dpi. At 3 dpi, no visible symptoms were observed. At 7 dpi, initial V-shaped lesions appeared along the leaf margin. At 10 dpi, V-shaped lesions expanded and vein blackening became evident. At 14 dpi, plants inoculated with *Xcc* 8004 alone developed severe disease symptoms, including V-shaped yellow lesions at the leaf margins and chlorosis (Figure 6A). No disease symptoms were observed in the negative control (CK) or in plants treated with phage X1 alone, confirming that the phage X1 alone did not cause visible symptoms under the conditions tested. Disease severity was significantly reduced in both co-treatment groups in which phage X1 was applied either therapeutically after infection (*Xcc* + X1) or preventively before infection (X1 + *Xcc*). Lesion lengths in the *Xcc* + X1 and X1 + *Xcc* groups were significantly reduced by 73.35 and 88.27%, respectively, compared with the *Xcc*-only treatment (*p* < 0.001, Figure 6B).

Enumeration of bacterial populations within plant tissues further supported these results (Figure 6C). The pathogen density in the *Xcc*-only control group reached 1.5 × 10^8^ CFU/g, whereas the densities in the *Xcc* + X1 and X1 + *Xcc* groups were reduced to 9.38 × 10^4^ CFU/g and 2.25 × 10^4^ CFU/g, respectively. The therapeutic and preventive treatments reduced culturable *Xcc* populations by approximately 3.20 and 3.82 log10 CFU/g, respectively, compared with the *Xcc*-only control (*p* < 0.001). Preventive application of phage X1 provided greater protection than therapeutic application. The pathogen population in the preventive treatment group (X1 + *Xcc*) was approximately fourfold lower than that in the therapeutic group (*Xcc* + X1), indicating that preventive phage treatment conferred superior protection against *Xcc* 8004 infection (*p* < 0.05).

## 4 Discussion

The exploration of phages as biocontrol agents has gained increasing attention due to their host specificity, environmental ubiquity, and ecological safety. In this study, we isolated a novel lytic phage X1, infecting *Xcc* 8004, from sediment samples of a karst cave. Phage X1 exhibits promising biological characteristics, a genomic architecture hypothesized to be associated with adaptation to oligotrophic environments, and significant biocontrol potential against cabbage black rot. These findings indicate that phage X1 represents a previously undescribed phage species within the class *Caudoviricetes* and may serve as a potential candidate for sustainable disease management. Furthermore, this work expands the diversity of known *Xcc*-specific phages and provides insight into phage-host interactions in nutrient-limited environments.

The biological properties of a phage are critical for its practical application as biocontrol agent. Phage X1 exhibited a narrow host range, infecting three tested *Xcc* strains but showing no lytic activity against the non-*Xcc* strains included in this study. This specificity may be advantageous for targeted pathogen suppression, although effects on plant associated microbial communities were not assessed in this study (Hyman and Abedon, 2010; Holtappels et al., 2022). The phage also exhibited rapid adsorption, with more than 99% of virions adsorbing to host cells within 10 min. Rapid adsorption is an important determinant of infection efficiency because it accelerates host recognition and initiation of the lytic cycle (Yehl et al., 2019). One-step growth curve analysis revealed a latent period of 60 min and a burst size of approximately 123 PFU per infected cell, indicating an efficient replication cycle capable of rapidly reducing bacterial populations.

Environmental stability is often a limiting factor in the field application of phage-based biocontrol agents (Halawa, 2023). Phage X1 exhibits stability across a wide temperature range (4 °C to 50 °C) and pH values (5 to 9), suggesting strong resilience to environmental fluctuations that may occur during storage, transport, and field application (Hu et al., 2023). Resistance to chloroform further suggests that the virions lack essential lipid components, confirming that X1 is a non-enveloped virus typical of phages within the class *Caudoviricetes* (Nakayinga et al., 2021). In addition, the phage X1 titer was maintained after prolonged UV exposure to UV-C (254 nm) and UV-A (365 nm), indicating tolerance to laboratory UV exposure conditions. However, the relationship between these results and persistence under natural sunlight conditions requires further investigation. Collectively, these stability profiles support the potential applicability of phage X1 under diverse environmental conditions.

Phage X1 possesses a relatively large genome (200,058 bp), compared with most previously reported *Xcc* phages (40 to 113 kb) (Erdrich et al., 2022; Holtappels et al., 2022; Neoralová et al., 2023; Evseev et al., 2024; Vique et al., 2025). Karst cave environments are typically oligotrophic, and the large genome may be consistent with adaptation to complete environmental pressures (Alexyuk et al., 2023; Liang et al., 2024), but this hypothesis remains speculative. In addition, the X1 genome encodes 34 tRNA genes corresponding to 18 amino acids, a feature commonly observed in large phage genomes. It has been reported that some large tailed phages encode multiple tRNA (Enav et al., 2012; Yoshikawa et al., 2018). However, the presence of numerous tRNAs in X1 does not imply a broad host range.

Phylogenetic and comparative genomic analyses confirmed that phage X1 is most closely related to *Xanthomonas* phage BUDD, although the two phages remain genetically distinct. Phylogenetic trees based on conserved genes revealed that X1 and BUDD formed a distinct clade that clusters with phages of the genus *Menderavirus* (infecting *Stenotrophomonas*). Genome-wide intergenomic similarity between X1 and BUDD reached 84.9%, whereas similarities with *Menderavirus* phages were substantially lower (17.0 to 17.5%). Because these related phages remain unclassified at the family and order level within the class *Caudoviricetes*, X1 represents a new species-level phage closely related to *Xanthomonas* phage BUDD within the class *Caudoviricetes*.

The genome of phage X1 was predicted to contain 28 putative AMGs, which are thought to mediate interactions between phages and host metabolic pathways (Huang et al., 2021; Yuan and Ju, 2023). These AMGs may be associated with host metabolic capacity during infection, thereby supporting efficient phage replication. Genes involved in amino acid metabolism (*TDH*, *serK*, *argG*, *asnB*, *GTK*) may expand the host amino acid pool, supporting the synthesis of progeny phage proteins (Huang et al., 2021). The presence of genes involved in NAD^+^ biosynthesis (*pncB*, *nadM*, *nadD*, *nadE*) may help maintain cellular redox balance during infection and potentially counteract host defense mechanisms involving NAD^+^ depletion (Osterman et al., 2024). Additional cofactor-related genes such as *coaE* and *thiF* may contribute to metabolic stability under environmental stress (Wang et al., 2022). Transport-related genes (*modF*, *tupC*, *nikE*, *wtpC*) may be associated with improved host acquisition of essential trace elements, thereby sustaining metabolic activity for phage replication (Eitinger and Mandrand-Berthelot, 2000; Self et al., 2001). Additionally, sulfate metabolism genes (*cysD*, *cysH*) may enhance assimilatory sulfate reduction, providing sulfur-containing amino acids for phage protein synthesis (Yuan and Ju, 2023). However, it is critical to note that the functionality of these computationally predicted AMGs remains to be verified.

Some AMGs may also be involved in host surface structures. Genes such as *gumH* and *wcaC,* involved in exopolysaccharide (EPS) biosynthesis may affect biofilm formation and bacterial pathogenicity (Biosca et al., 2021; Tang et al., 2024). Genes involved in lipopolysaccharide or cell wall modification (*lpxH*, *tagE*, and *tarM*) may influence phage adsorption efficiency because these structures frequently function as phage receptors (Hyman and Abedon, 2010). The coexistence of these genes with the high adsorption efficiency observed for phage X1 may indicate complex phage-host interactions; however, direct functional relationships remain to be experimentally verified.

The biocontrol potential of phage X1 was validated *in planta* using cabbage seedlings. Preventive and therapeutic treatments significantly reduced disease symptoms, decreasing lesion lengths by approximately 88 and 73%, respectively. These reductions are greater than those reported for some previously described *Xcc* phages and comparable to others including ɸEF1 and FoX2 (Papaianni et al., 2020; Holtappels et al., 2022; Vique et al., 2025). Preventive application proved more effective than therapeutic treatment, consistent with previous studies (Holtappels et al., 2022; Vique et al., 2025). This effect is likely due to the establishment of a protective phage population on the leaf surfaces that can rapidly lyse invading pathogen cells before the infection becomes established (Jones et al., 2007; Buttimer et al., 2017). Once pathogens penetrate the internal tissues of plants, physical barriers such as plant cell walls may limit phage access to the pathogen (Lee S. et al., 2021). These findings support preventive phage application as an effective strategy for controlling *Xcc*-induced black rot.

Despite these promising results, several limitations remain. First, the biocontrol efficacy was only evaluated under controlled pot culture conditions, and field trials are required to verify performance under diverse agricultural environments. Furthermore, this study only evaluated the direct biocontrol phenotype of phage X1; the physiological, biochemical, and molecular responses of *Brassica* to *Xcc* and phage X1 will be explored in future research. Second, the narrow host range of phage X1 may limit its effectiveness against genetically diverse *Xcc* field populations. These limitations restrict the interpretation of X1 as a practical biocontrol agent at this stage. Third, the predicted functions of the 28 AMGs require experimental validation using methods such as gene knockout or heterologous expression. Finally, formulation strategies, including microcapsulation or protective additives, may improve the persistence and shelf life of phage X1 under field conditions (Zhang et al., 2025).

In conclusion, this study characterized a large-genome lytic bacteriophage, X1, isolated from an oligotrophic karst cave. Its favorable biological properties, distinctive genomic features, including abundant tRNA genes and a diverse repertoire of putative AMGs, and significant biocontrol efficacy against cabbage black rot highlight its potential as a candidate for further development as a phage-based biocontrol agent.

## Data Availability

The raw data generated in this study can be found in the NCBI GenBank, under accession OQ427096.1: https://www.ncbi.nlm.nih.gov/datasets/genome/GCA_029376345.1.

## Footnotes

1
http://topaz.gatech.edu/GeneMark/genemarks.cgi

2
http://rast.nmpdr.org/rast.cgi

3
https://blast.ncbi.nlm.nih.gov/Blast

4
http://lowelab.ucsc.edu/tRNAscan-SE/

5
https://proksee.ca/

## References

[ref1] Alcock B. P. Huynh W. Chalil R. Smith K. W. Raphenya A. R. Wlodarski M. A. . (2023). CARD 2023: expanded curation, support for machine learning, and resistome prediction at the comprehensive antibiotic resistance database. Nucleic Acids Res. 51, D690–d699. doi: 10.1093/nar/gkac920, 36263822 PMC9825576

[ref2] Alengebawy A. Abdelkhalek S. T. Qureshi S. R. Wang M. Q. (2021). Heavy metals and pesticides toxicity in agricultural soil and plants: ecological risks and human health implications. Toxics 9:42. doi: 10.3390/toxics9030042, 33668829 PMC7996329

[ref3] Alexyuk P. Bogoyavlenskiy A. Alexyuk M. Akanova K. Moldakhanov Y. Berezin V. (2023). Isolation and characterization of jumbo coliphage vB_EcoM_Lh1B as a promising therapeutic agent against chicken colibacillosis. Microorganisms 11, 1524–1543. doi: 10.3390/microorganisms11061524, 37375026 PMC10302289

[ref4] Altschul S. F. Gish W. Miller W. Myers E. W. Lipman D. J. (1990). Basic local alignment search tool. J. Mol. Biol. 215, 403–410. doi: 10.1016/s0022-2836(05)80360-2, 2231712

[ref5] Bankevich A. Nurk S. Antipov D. Gurevich A. A. Dvorkin M. Kulikov A. S. . (2012). SPAdes: a new genome assembly algorithm and its applications to single-cell sequencing. J. Comput. Biol. 19, 455–477. doi: 10.1089/cmb.2012.0021, 22506599 PMC3342519

[ref6] Bernard C. Labreuche Y. Diarra C. Daszkowski P. Cahier K. Goudenège D. . (2025). Adaptive genomic plasticity in large-genome, broad-host-range vibrio phages. ISME J. 19:wraf063. doi: 10.1093/ismejo/wraf063, 40184633 PMC12028318

[ref7] Biosca E. G. Català-Senent J. F. Figàs-Segura À. Bertolini E. López M. M. Álvarez B. (2021). Genomic analysis of the first european bacteriophages with depolymerase activity and biocontrol efficacy against the phytopathogen *Ralstonia solanacearum*. Viruses 13, 2539–2559. doi: 10.3390/v13122539, 34960808 PMC8703784

[ref8] Buttimer C. McAuliffe O. Ross R. P. Hill C. O'Mahony J. Coffey A. (2017). Bacteriophages and bacterial plant diseases. Front. Microbiol. 8, 34–49. doi: 10.3389/fmicb.2017.00034, 28163700 PMC5247434

[ref9] Coil D. Jospin G. Darling A. E. (2015). A5-miseq: an updated pipeline to assemble microbial genomes from Illumina MiSeq data. Bioinformatics 31, 587–589. doi: 10.1093/bioinformatics/btu661, 25338718

[ref10] de Leeuw M. Baron M. Ben David O. Kushmaro A. (2020). Molecular insights into bacteriophage evolution toward its host. Viruses 12, 1132–1144. doi: 10.3390/v12101132, 33036277 PMC7599783

[ref11] Dong Y. Gao J. Wu Q. Ai Y. Huang Y. Wei W. . (2020). Co-occurrence pattern and function prediction of bacterial community in karst cave. BMC Microbiol. 20, 137–150. doi: 10.1186/s12866-020-01806-7, 32471344 PMC7257168

[ref12] Eitinger T. Mandrand-Berthelot M. A. (2000). Nickel transport systems in microorganisms. Arch. Microbiol. 173, 1–9. doi: 10.1007/s002030050001, 10648098

[ref13] Enav H. Béjà O. Mandel-Gutfreund Y. (2012). Cyanophage tRNAs may have a role in cross-infectivity of oceanic *Prochlorococcus* and *Synechococcus* hosts. ISME J. 6, 619–628. doi: 10.1038/ismej.2011.146, 22011720 PMC3280135

[ref14] Erdrich S. H. Sharma V. Schurr U. Arsova B. Frunzke J. (2022). Isolation of novel *Xanthomonas* phages infecting the plant pathogens *X. Translucens* and *X. campestris*. Viruses 14:1449. doi: 10.3390/v14071449, 35891434 PMC9316219

[ref15] Evseev P. V. Tarakanov R. I. Vo H. T. N. Suzina N. E. Vasilyeva A. A. Ignatov A. N. . (2024). Characterisation of new Foxunavirus phage Murka with the potential of *Xanthomonas campestris* pv. *Campestris* control. Viruses 16, 18–29. doi: 10.3390/v16020198, 38399973 PMC10892653

[ref16] Fujiki J. Yoshida S. I. Nakamura T. Nakamura K. Amano Y. Nishida K. . (2021). Novel virulent bacteriophage ΦSG005, which infects *Streptococcus gordonii*, forms a distinct clade among *Streptococcus* viruses. Viruses 13, 1964–1976. doi: 10.3390/v13101964, 34696394 PMC8537203

[ref17] Greer S. F. Surendran A. Grant M. Lillywhite R. (2023). The current status, challenges, and future perspectives for managing diseases of brassicas. Front. Microbiol. 14:1209258. doi: 10.3389/fmicb.2023.1209258, 37533829 PMC10392840

[ref18] Halawa E. M. (2023). Challenges of bacteriophages application in controlling bacterial plant diseases and how to overcome them. J. Genet. Eng. Biotechnol. 21, 98–107. doi: 10.1186/s43141-023-00549-y, 37815601 PMC10564689

[ref19] Han J. R. Li S. Li W. J. Dong L. (2024). Mining microbial and metabolic dark matter in extreme environments: a roadmap for harnessing the power of multi-omics data. Adv Biotechnol (Singap) 2, 26–44. doi: 10.1007/s44307-024-00034-8, 39883228 PMC11740847

[ref20] Holtappels D. Fortuna K. J. Moons L. Broeckaert N. Bäcker L. E. Venneman S. . (2022). The potential of bacteriophages to control *Xanthomonas campestris* pv. *Campestris* at different stages of disease development. Microb. Biotechnol. 15, 1762–1782. doi: 10.1111/1751-7915.14004, 35084112 PMC9151335

[ref21] Hu M. Xing B. Yang M. Han R. Pan H. Guo H. . (2023). Characterization of a novel genus of jumbo phages and their application in wastewater treatment. iScience 26:106947. doi: 10.1016/j.isci.2023.106947, 37324530 PMC10265529

[ref22] Huang X. Jiao N. Zhang R. (2021). The genomic content and context of auxiliary metabolic genes in roseophages. Environ. Microbiol. 23, 3743–3757. doi: 10.1111/1462-2920.15412, 33511765

[ref23] Hyman P. Abedon S. T. (2010). Bacteriophage host range and bacterial resistance. Adv. Appl. Microbiol. 70, 217–248. doi: 10.1016/s0065-2164(10)70007-1, 20359459

[ref24] Jain L. Kumar V. Jain S. K. Kaushal P. Ghosh P. K. (2023). Isolation of bacteriophages infecting *Xanthomonas oryzae* pv. *Oryzae* and genomic characterization of novel phage vB_XooS_NR08 for biocontrol of bacterial leaf blight of rice. Front. Microbiol. 14:1084025. doi: 10.3389/fmicb.2023.1084025, 37007514 PMC10061587

[ref25] Jones J. B. Jackson L. E. Balogh B. Obradovic A. Iriarte F. B. Momol M. T. (2007). Bacteriophages for plant disease control. Annu. Rev. Phytopathol. 45, 245–262. doi: 10.1146/annurev.phyto.45.062806.094411, 17386003

[ref26] Khan Mirzaei M. Nilsson A. S. (2015). Isolation of phages for phage therapy: a comparison of spot tests and efficiency of plating analyses for determination of host range and efficacy. PLoS One 10:e0118557. doi: 10.1371/journal.pone.0118557, 25761060 PMC4356574

[ref9001] Kumar S. Stecher G. Tamura K. (2016). MEGA7: molecular evolutionary genetics analysis version 7.0 for bigger datasets. Mol. Biol. Evol. 33, 1870–1874. doi: 10.1093/molbev/msw054 27004904 PMC8210823

[ref27] Kurtz S. Phillippy A. Delcher A. L. Smoot M. Shumway M. Antonescu C. . (2004). Versatile and open software for comparing large genomes. Genome Biol. 5:R12. doi: 10.1186/gb-2004-5-2-r12, 14759262 PMC395750

[ref28] Lee S. Y. Thapa Magar R. Kim H. J. Choi K. Lee S. W. (2021). Complete genome sequence of a novel bacteriophage RpY1 infecting *Ralstonia solanacearum* strains. Curr. Microbiol. 78, 2044–2050. doi: 10.1007/s00284-021-02466-0, 33835234

[ref29] Lee S. Vu N. T. Oh E. J. Rahimi-Midani A. Thi T. N. Song Y. R. . (2021). Biocontrol of soft rot caused by *Pectobacterium odoriferum* with bacteriophage phiPccP-1 in kimchi cabbage. Microorganisms 9, 779–795. doi: 10.3390/microorganisms9040779, 33917817 PMC8068257

[ref30] Liang J. L. Feng S. W. Lu J. L. Wang X. N. Li F. L. Guo Y. Q. . (2024). Hidden diversity and potential ecological function of phosphorus acquisition genes in widespread terrestrial bacteriophages. Nat. Commun. 15, 2827–2842. doi: 10.1038/s41467-024-47214-7, 38565528 PMC10987575

[ref31] Liang X. Yang S. Radosevich M. Wang Y. Duan N. Jia Y. (2025). Bacteriophage-driven microbial phenotypic heterogeneity: ecological and biogeochemical importance. NPJ Biofilms Microbiomes 11, 82–94. doi: 10.1038/s41522-025-00727-5, 40399330 PMC12095545

[ref32] Liu Z. Wang H. Wang J. Lv J. Xie B. Luo S. . (2022). Physical, chemical, and biological control of black rot of brassicaceae vegetables: a review. Front. Microbiol. 13:1023826. doi: 10.3389/fmicb.2022.1023826, 36504826 PMC9726911

[ref33] Liu B. Zheng D. Jin Q. Chen L. Yang J. (2019). VFDB 2019: a comparative pathogenomic platform with an interactive web interface. Nucleic Acids Res. 47, D687–d692. doi: 10.1093/nar/gky1080, 30395255 PMC6324032

[ref34] Moraru C. Varsani A. Kropinski A. M. (2020). VIRIDIC-A novel tool to calculate the intergenomic similarities of prokaryote-infecting viruses. Viruses 12, 1268–1278. doi: 10.3390/v12111268, 33172115 PMC7694805

[ref35] Nakayinga R. Makumi A. Tumuhaise V. Tinzaara W. (2021). *Xanthomonas* bacteriophages: a review of their biology and biocontrol applications in agriculture. BMC Microbiol. 21, 291–306. doi: 10.1186/s12866-021-02351-7, 34696726 PMC8543423

[ref36] Neoralová M. Brázdová S. Eichmeier A. Petrzik K. (2023). Complete genome sequence of *Xanthomonas* phage M29, a new member of Foxunavirus isolated in the Czech Republic. Virus Genes 59, 874–877. doi: 10.1007/s11262-023-02027-6, 37667026

[ref37] Nishimura Y. Yoshida T. Kuronishi M. Uehara H. Ogata H. Goto S. (2017). ViPTree: the viral proteomic tree server. Bioinformatics 33, 2379–2380. doi: 10.1093/bioinformatics/btx157, 28379287

[ref38] Osterman I. Samra H. Rousset F. Loseva E. Itkin M. Malitsky S. . (2024). Phages reconstitute NAD(+) to counter bacterial immunity. Nature 634, 1160–1167. doi: 10.1038/s41586-024-07986-w, 39322677

[ref39] Papaianni M. Paris D. Woo S. L. Fulgione A. Rigano M. M. Parrilli E. . (2020). Plant dynamic metabolic response to bacteriophage treatment after *Xanthomonas campestris* pv. *Campestris* infection. Front. Microbiol. 11:732. doi: 10.3389/fmicb.2020.00732, 32390981 PMC7189621

[ref40] Pratama A. A. Pérez-Carrascal O. Sullivan M. B. Küsel K. (2026). Diversity and ecological roles of hidden viral players in groundwater microbiomes. Nat. Commun. 17:2179. doi: 10.1038/s41467-026-68914-2, 41617723 PMC12960796

[ref41] Ramnarine S. Jayaraman J. Ramsubhag A. (2024). Crucifer lesion-associated *Xanthomonas* strains show multi-resistance to heavy metals and antibiotics. Curr. Microbiol. 81, 136–149. doi: 10.1007/s00284-024-03646-4, 38598029

[ref42] Sabri M. El Handi K. El Tousy A. De Stradis A. Elbeaino T. (2024). Synergistic antibacterial activity of *Lactococcus lactis* and *Xylella* phage MATE 2 for an effective biocontrol strategy against black rot disease in broccoli. Front. Microbiol. 15:1468792. doi: 10.3389/fmicb.2024.1468792, 39224218 PMC11366581

[ref43] Savary S. Willocquet L. Pethybridge S. J. Esker P. McRoberts N. Nelson A. (2019). The global burden of pathogens and pests on major food crops. Nat Ecol Evol 3, 430–439. doi: 10.1038/s41559-018-0793-y, 30718852

[ref44] Schubert M. Lindgreen S. Orlando L. (2016). AdapterRemoval v2: rapid adapter trimming, identification, and read merging. BMC. Res. Notes 9, 88–101. doi: 10.1186/s13104-016-1900-2, 26868221 PMC4751634

[ref45] Self W. T. Grunden A. M. Hasona A. Shanmugam K. T. (2001). Molybdate transport. Res. Microbiol. 152, 311–321. doi: 10.1016/s0923-2508(01)01202-5, 11421278

[ref46] Shaffer M. Borton M. A. McGivern B. B. Zayed A. A. La Rosa S. L. Solden L. M. . (2020). DRAM for distilling microbial metabolism to automate the curation of microbiome function. Nucleic Acids Res. 48, 8883–8900. doi: 10.1093/nar/gkaa621, 32766782 PMC7498326

[ref47] Skliros D. Papazoglou P. Gkizi D. Paraskevopoulou E. Katharios P. Goumas D. E. . (2023). In planta interactions of a novel bacteriophage against *Pseudomonas syringae* pv. *Tomato*. Appl. Microbiol. Biotechnol. 107, 3801–3815. doi: 10.1007/s00253-023-12493-5, 37074382 PMC10175458

[ref48] Stefani E. Obradović A. Gašić K. Altin I. Nagy I. K. Kovács T. (2021). Bacteriophage-mediated control of phytopathogenic *Xanthomonads*: a promising green solution for the future. Microorganisms 9, 1056–1069. doi: 10.3390/microorganisms9051056, 34068401 PMC8153558

[ref49] Sullivan M. J. Petty N. K. Beatson S. A. (2011). Easyfig: a genome comparison visualizer. Bioinformatics 27, 1009–1010. doi: 10.1093/bioinformatics/btr039, 21278367 PMC3065679

[ref50] Tang Z. Xu H. Xiao H. Zhu R. Li D. Zhao Z. . (2024). Different nitrogen conditions regulating extracellular polymeric substances and erosion resistance of sewer sediment: mechanism of microbial metabolism and molecular response. Chemosphere 368:143661. doi: 10.1016/j.chemosphere.2024.143661, 39510270

[ref51] Turner D. Kropinski A. M. Adriaenssens E. M. (2021). A roadmap for genome-based phage taxonomy. Viruses 13, 506–519. doi: 10.3390/v13030506, 33803862 PMC8003253

[ref52] Ulbrich J. Jobe N. E. Jones D. S. Kieft T. L. (2024). Cave pools in Carlsbad caverns national park contain diverse bacteriophage communities and novel viral sequences. Microb. Ecol. 87, 163–178. doi: 10.1007/s00248-024-02479-9, 39724159 PMC11671562

[ref53] Vicente J. G. Holub E. B. (2013). *Xanthomonas campestris* pv. *Campestris* (cause of black rot of crucifers) in the genomic era is still a worldwide threat to brassica crops. Mol. Plant Pathol. 14, 2–18. doi: 10.1111/j.1364-3703.2012.00833.x, 23051837 PMC6638727

[ref54] Vique G. Mendoza-Barberá E. Ramos-Barbero M. D. Blanco-Picazo P. Sala-Comorera L. Quirós P. . (2025). Efficacy of *Erwinia amylovora* and *Xanthomonas campestris* pv *campestris* phages to control fire blight and black rot in vivo. Microbiol. Spectrum 13:e0028025. doi: 10.1128/spectrum.00280-25, 40377312 PMC12211020

[ref55] Walker B. J. Abeel T. Shea T. Priest M. Abouelliel A. Sakthikumar S. . (2014). Pilon: an integrated tool for comprehensive microbial variant detection and genome assembly improvement. PLoS One 9:e112963. doi: 10.1371/journal.pone.0112963, 25409509 PMC4237348

[ref56] Wang Q. Cai L. Zhang R. Wei S. Li F. Liu Y. . (2022). A unique set of auxiliary metabolic genes found in an isolated cyanophage sheds new light on marine phage-host interactions. Microbiol. Spectrum 10:e0236722. doi: 10.1128/spectrum.02367-22, 36190421 PMC9602691

[ref57] Wang C. Zheng R. Zhang T. Sun C. (2024). Polysaccharides induce deep-sea Lentisphaerae strains to release chronic bacteriophages. eLife 13:RP92345. doi: 10.7554/eLife.92345, 39207920 PMC11361711

[ref58] Wu Q. An N. Fang Z. Li S. Xiang L. Liu Q. . (2024). Characteristics and whole-genome analysis of a novel *Pseudomonas syringae* pv. *Tomato* bacteriophage D6 isolated from a karst cave. Virus Genes 60, 295–308. doi: 10.1007/s11262-024-02064-9, 38594490 PMC11139720

[ref59] Yang M. Chen H. Huang Q. Xie Z. Liu Z. Zhang J. . (2022). Characterization of the novel phage vB_VpaP_FE11 and its potential role in controlling *Vibrio parahaemolyticus* biofilms. Viruses 14, 264–277. doi: 10.3390/v14020264, 35215857 PMC8879856

[ref60] Yehl K. Lemire S. Yang A. C. Ando H. Mimee M. Torres M. T. . (2019). Engineering phage host-range and suppressing bacterial resistance through phage tail fiber mutagenesis. Cell 179, 459–469.e9. doi: 10.1016/j.cell.2019.09.015, 31585083 PMC6924272

[ref61] Yoshikawa G. Askora A. Blanc-Mathieu R. Kawasaki T. Li Y. Nakano M. . (2018). *Xanthomonas citri* jumbo phage XacN1 exhibits a wide host range and high complement of tRNA genes. Sci. Rep. 8, 4486–4496. doi: 10.1038/s41598-018-22239-3, 29540765 PMC5852040

[ref62] Yuan L. Ju F. (2023). Potential auxiliary metabolic capabilities and activities reveal biochemical impacts of viruses in municipal wastewater treatment plants. Environ. Sci. Technol. 57, 5485–5498. doi: 10.1021/acs.est.2c07800, 36947091

[ref63] Zhang S. Ryu K. Kim J. C. Ahn J. (2025). Characterization and application of novel bacteriophage PS2 for controlling pathogenic *Escherichia coli* in different food matrices. Int. J. Food Microbiol. 441:111330. doi: 10.1016/j.ijfoodmicro.2025.111330, 40602192

