# Supplementary material for: Genomic and biological characterization of a novel bacteriophage X1 infecting Xanthomonas campestris pv. campestris with biocontrol potential against cabbage black rot
Source: Front Microbiol. 2026 Jul 7;17:1827519. doi: 10.3389/fmicb.2026.1827519 (PMC13384950; doi:10.3389/fmicb.2026.1827519)
Supplement: Supplementary file 1 [file Data_Sheet_1.pdf]

Supplementary Material

1 Supplementary Tables

Supplementary Table S1. List of bacterial strains and their sensitivity to phage X1.

Bacterial strain	Sensitivity to X1	Source	EOP
Xanthomonas campestris pv. *campestris* 8004	+	*Brassica oleracea var. botrytis*	1
Xanthomonas campestris pv. *campestris* XC1	+	*Brassica oleracea*	0.27 ± 0.01
Xanthomonas campestris pv. *campestris* ATCC 33913	+	*Brassica oleracea var. gemmifera*	0.23 ± 0.01
Xanthomonas campestris pv. *badrii* ATCC 11672	—	*Xanthium strumarium*	—
Xanthomonas oryzae pv. *oryzae* 2212	—	*Oryza sativa*	—
Pseudomonas syringae pv. *tomato* DC3000	—	*Solanum lycopersicum*	—

+: indicates that the phage can infect the strain, –: indicates that the ophage cannot infect the strain.

The efficiency of plating (EOP) was calculated as the ratio of the mean plaque-forming units (PFU) obtained on test strains to the mean PFU on the propagating host. All values are presented as the mean ± standard deviation from three independent replicates. Phages with EOP ≥ 0.5 were considered as phages with high sensitivity, phages with 0.1 ≤ EOP ≤ 0.5 as phages with moderate sensitivity, and phages with 0.001 ≤ EOP ≤ 0.1 as phages with low sensitivity.

Supplementary Table S2. Predicted the known functional proteins in phage X1.

Group	ORF	Start	Stop	strand	Predicted function	Evalue	Identity（%）	Accession
Structural	1	821	1360	+	tail tube associated base plate protein	3E-138	96.45	WEM34222.1
	2	1357	2586	+	baseplate wedge protein	7E-117	90.50	WEM34223.1
	35	27003	28772	+	baseplate wedge protein	0	92.87	WEM34256.1
	36	28769	30265	+	baseplate wedge protein	0	93.98	WEM34257.1
	37	30277	31800	+	baseplate wedge protein	0	95.46	WEM34258.1
	41	37945	38709	+	neck protein	1E-169	94.49	WEM34262.1
	42	38744	39388	+	neck protein	5E-152	95.79	WEM34263.1
	43	39388	40128	+	tail assembly protein	9E-176	96.75	WEM34264.1
	47	43139	45115	+	tail sheath protein	0	94.53	WEM34268.1
	49	45742	46290	+	tail tube protein	4E-129	98.35	WEM34270.1
	50	46373	48022	+	portal head vertex protein	0	91.45	WEM34271.1
	51	48035	48829	+	putative prohead core protein	5E-176	90.15	WEM34272.1
	53	49096	49785	+	prohead core protein	5E-150	91.11	WEM34274.1
	54	49851	50663	+	prohead assembly （scaffolding） protein	2E-151	91.27	WEM34275.1
	103	84546	85328	+	baseplate hub protein	2E-165	91.15	WEM34324.1
	247	166965	167375	+	minor tail protein	2E-69	84.56	WEM34468.1
	281	182655	186770	+	tail fiber protein	0	85.72	WEM34502.1
	309	198815	198177	-	baseplate wedge protein	6E-147	97.07	WEM34530.1
	311	199876	199421	-	head completion protein	7E-104	94.04	WEM34532.1
Packaging	46	41420	43069	+	terminase large subunit	0	96.36	WEM34267.1
	55	50734	51969	+	major capsid protein	0	97.32	WEM34276.1
DNA replication and metabolism	57	52617	53162	+	deoxynucleoside monophosphate kinase	2E-125	94.48	WEM34278.1
	59	53727	54065	+	single stranded DNA-binding protein	3E-64	86.61	WEM34280.1
	60	54080	55579	+	DNA helicase	0	94.59	WEM34281.1
	62	56015	56494	+	RNA polymerase sigma factor	8E-114	98.11	WEM34283.1
	63	56504	57565	+	Endonuclease	0	99.14	WEM34284.1
	65	57777	59495	+	Endonuclease	0	94.41	WEM34286.1
	68	59971	60609	+	DNA polymerase sliding clamp	6E-147	98.11	WEM34289.1
	70	61052	61996	+	DNA polymerase accessory protein sliding clamp	0	98.70	WEM34291.1
	71	62014	62418	+	DNA polymerase clamp loader subunit	2E-93	98.51	WEM34292.1
	72	62387	62812	+	translation repressor protein	2E-94	93.62	WEM34293.1
	73	62799	63410	+	deoxyuridine triphosphatase	4E-129	96.77	WEM34294.1
	76	64041	66719	+	DNA polymerase	0	96.63	WEM34297.1
	78	67081	68130	+	RecA-like DNA recombinase	0	94.74	WEM34299.1
	86	72861	74174	+	queuine tRNA-ribosyltransferase	0	98.17	WEM34307.1
	93	76582	77985	+	DNA primase/helicase	0	94.86	WEM34314.1
	109	89224	90252	+	DNA primase	0	95.90	WEM34330.1
	113	91363	92226	+	ribonuclease H	0	95.04	WEM34334.1
	116	92759	93415	+	Exonuclease	5E-148	91.32	WEM34337.1
	121	99768	100403	+	Exonuclease	3E-154	98.10	WEM34342.1
	170	124040	126358	+	ribonucleotide reductase, alpha subunit	0	96.11	WEM34391.1
	171	126503	127591	+	ribonucleotide reductase, beta subunit	0	95.58	WEM34392.1
	172	127569	127958	+	zinc-finger-containing protein	2E-46	92.41	WEM34393.1
	180	131784	133073	+	DNA ligase	0	91.65	WEM34401.1
	186	134605	135087	+	nucleoside 2-deoxyribosyltransferase domain-containing protein	2E-107	93.12	WEM34407.1
	210	149672	150448	+	metallophosphoesterase	1E-161	93.83	WEM34431.1
	218	153463	153966	+	Metallophosphatase	1E-118	98.80	WEM34439.1
	221	154883	156745	+	DNA topoisomerase II large subunit	0	97.26	WEM34442.1
	222	156742	158106	+	DNA topoisomerase II medium subunit	0	96.26	WEM34443.1
	230	160402	161289	+	polynucleotide kinase	0	93.92	WEM34451.1
	249	167562	167924	+	nucleoside triphosphate pyrophosphohydrolase	4E-70	84.17	WEM34470.1
	254	169276	170247	+	thymidylate synthetase	0	91.30	WEM34475.1
	266	174623	175102	+	pyrimidine dimer DNA glycosylase/endonuclease	4E-108	91.82	WEM34487.1
	290	190283	191398	+	RNA ligase	0	76.82	WEM34511.1
	305	195579	196652	+	multifunctional CCA addition/repair protein	0	72.88	WEM34526.1
	307	197567	197097	-	Endonuclease	1E-107	94.87	WEM34528.1
	310	199417	198812	-	DNA end protector protein	3E-145	98.01	WEM34531.1
biosynthesis	13	8840	8034	-	ATP-grasp domain-containing protein	2E-179	87.31	WEM34234.1
	14	10273	8909	-	SPFH domain-containing protein	0	91.85	WEM34236.1
	29	19097	21496	+	LamG domain-containing protein	0	85.50	WEM34250.1

Group	ORF	Start	Stop	strand	Predicted function	Evalue	Identity（%）	Accession
	33	26369	26617	+	PAAR motif of membran proteins	1E-29	65.06	WEM34254.1
	81	69405	70088	+	GTP cyclohydrolase 1	1E-149	95.59	WEM34302.1
	82	70090	70623	+	phosphoadenosine phosphosulfate reductase family protein	2E-97	86.59	WEM34303.1
	88	74616	75509	+	QueE-like queosine biosynthesis protein	0	98.65	WEM34309.1
	99	82528	83016	+	adenylyltransferase/cytidyltransferase family protein	7E-102	87.65	WEM34320.1
	120	95507	99694	+	ADP-ribosyltransferase	0	87.61	WEM34341.1
	137	106150	106875	+	2OG-Fe（II） oxygenase	5E-151	85.06	WEM34358.1
	138	106879	107709	+	NAD（+） synthase	1E-177	88.04	WEM34359.1
	141	109627	110676	+	nicotinamide-nucleotide adenylyltransferase	0	91.45	WEM34362.1
	143	111193	112638	+	nicotinamide phosphoribosyltransferase	0	90.64	WEM34364.1
	145	113058	114374	+	Glycosyltransferase	0	95.49	WEM34366.1
	159	119034	119561	+	Phosphohydrolase	2E-123	97.13	WEM34380.1
	173	127951	128175	+	Glutaredoxin	1E-45	97.30	WEM34394.1
	193	142018	142266	+	GroES molecular chaperone protein	7E-46	90.24	WEM34414.1
	235	163127	163924	+	SPFH domain-containing protein	7E-100	57.36	WEM34456.1
	261	172197	172703	+	6-pyruvoyl tetrahydrobiopterin synthase	1E-114	94.67	WEM34482.1
Lysis	288	189416	190012	+	cell wall hydrolase	4E-138	93.43	WEM34509.1
	234	162878	163123	+	transmembrane protein	3E-28	65.43	WEM34455.1

Supplementary Table S3. Conserved domains found in ORFs of the phage X1 genome.

ORF	From	To	Short name	E-Value	Bitscore	Accession
1	36	176	Phage_gp53 superfamily	7.58E-11	57.80	cl26475
3	9	448	5 superfamily	2.04E-68	228.49	cl33691
7	13	142	DUF4262 superfamily	1.10E-19	78.85	cl16597
13	125	256	R2K_2 superfamily	1.30E-13	67.05	cl23655
14	60	210	Band_7	3.72E-14	70.43	pfam01145
14	74	443	YqiK superfamily	7.56E-12	67.16	cl34451
24	364	524	LamG superfamily	3.19E-10	58.93	cl22861
24	160	281	LamG superfamily	4.66E-06	46.61	cl22861
28	92	202	WD40	0.0013246	40.84	COG2319
29	329	460	Laminin_G_3	1.22E-16	77.42	pfam13385
29	623	783	Laminin_G_3	2.02E-14	71.26	pfam13385
29	113	233	Laminin_G_3	2.29E-13	68.18	pfam13385
33	2	82	PAAR_1	7.03E-27	93.50	cd14737
34	1	126	GPW_gp25 superfamily	2.30E-13	61.94	cl01403
35	1	582	Baseplate_J superfamily	4.44E-101	318.91	cl01294
36	89	114	7 superfamily	8.28E-06	48.57	cl14354
37	82	233	Phage-Gp8 superfamily	4.83E-06	48.58	cl27861
38	776	1001	DUF4815 superfamily	6.09E-24	107.85	cl24594
38	11	377	DUF4815 superfamily	6.45E-21	98.22	cl24594
38	566	846	COG1361 superfamily	0.0086634	39.85	cl27848
41	1	249	13 superfamily	3.42E-57	184.57	cl14347
42	1	214	T4_neck-protein superfamily	3.85E-46	151.71	cl27828
43	5	225	T4-gp15_tss superfamily	1.75E-44	150.59	cl14348
45	37	139	DNA_Packaging superfamily	1.34E-05	42.84	cl27835
46	12	515	17 superfamily	0	621.30	cl28557
47	6	655	18 superfamily	6.84E-144	433.34	cl33682
49	9	176	19 superfamily	2.39E-40	134.02	cl28641
50	58	499	Peptidase_S80	3.24E-176	504.03	pfam07230
53	13	197	Peptidase_S77 superfamily	2.99E-49	160.25	cl11614
54	55	225	22 superfamily	1.36E-07	51.26	cl20173
55	11	410	Gp23 superfamily	3.63E-158	455.81	cl22495
57	4	178	NK superfamily	5.72E-23	91.30	cl17190
58	9	157	3 superfamily	9.70E-23	88.60	cl24094
59	1	111	UvsY superfamily	1.70E-16	69.29	cl12619
60	3	498	uvsW superfamily	0	529.58	cl33684
62	3	120	55 superfamily	1.10E-29	106.33	cl14345
63	1	346	47 superfamily	5.11E-92	278.42	cl26377
65	1	572	46 superfamily	5.67E-144	427.51	cl33686
66	41	70	Glyco_tranf_GTA_type superfamily	0.0059144	33.13	cl11394
68	1	212	45 superfamily	7.74E-51	164.01	cl31814
70	1	314	44 superfamily	7.80E-109	318.86	cl33683
71	7	121	Phage_clamp_A superfamily	1.23E-15	69.36	cl27778
72	15	135	Translat_reg superfamily	5.29E-41	132.84	cl28116
73	45	203	trimeric_dUTPase superfamily	8.59E-39	130.28	cl00493
76	1	892	43 superfamily	0	679.49	cl31813
81	61	227	folE	2.68E-72	218.11	PRK09347
82	3	172	CysH superfamily	4.40E-22	89.41	cl43048
85	215	366	Glycosyltransferase_GTB-type superfamily	3.74E-11	60.75	cl10013
88	6	262	NrdG superfamily	1.11E-12	65.87	cl33971
93	7	433	41 superfamily	7.60E-175	498.82	cl29348
95	9	49	AbiEi_4	0.0035223	34.82	pfam13338
99	6	131	nt_trans superfamily	1.11E-08	50.78	cl00015
101	239	268	DUF5710 superfamily	2.69E-08	49.11	cl44662
101	117	265	PTZ00121 superfamily	0.0001273	43.59	cl31754
102	53	108	ParB_N_Srx superfamily	1.32E-05	40.61	cl28891
103	115	259	T4_baseplate superfamily	5.75E-06	44.60	cl44379
105	501	646	Lyz-like superfamily	6.24E-06	46.39	cl00222
105	299	345	FIX-like superfamily	0.0006181	38.67	cl41761
109	3	337	61 superfamily	5.16E-87	265.32	cl26791
110	47	226	P-loop_NTPase superfamily	2.08E-32	116.81	cl38936
113	1	282	rnh superfamily	1.14E-76	235.72	cl33687
114	1	44	CxxC_CXXC_SSSS	4.34E-08	44.47	smart00834
118	14	193	59 superfamily	2.88E-14	68.52	cl33685
119	3	298	gp32 superfamily	1.16E-99	294.67	cl17537
120	1163	1248	ADP_ribosyl superfamily	9.10E-10	57.71	cl00283
121	5	188	DnaQ_like_exo superfamily	2.62E-36	126.72	cl10012
122	55	80	Peptidase_C39_like superfamily	0.005613	34.55	cl00296
125	51	75	P-loop_NTPase superfamily	0.0016447	34.91	cl38936
126	28	105	Oxidoreductase_nitrogenase superfamily	0.0084753	34.18	cl02775
130	6	42	PLN00175 superfamily	0.0029262	33.68	cl30195
136	45	109	cyt_tran_rel	4.56E-06	43.83	TIGR00125
137	122	232	2OG-FeII_Oxy_3	3.15E-06	44.29	pfam13640
138	31	265	AANH_like superfamily	1.05E-38	135.75	cl00292

ORF	From	To	Short name	E-Value	Bitscore	Accession
141	1	348	PRK05379 superfamily	9.52E-116	338.91	cl28366
143	1	439	PRK09198	0	600.73	PRK09198
145	16	372	Glycosyltransferase_GTB-type superfamily	0.0001735	43.30	cl10013
155	15	40	DS superfamily	0.0049968	33.87	cl00826
155	25	72	POLBc superfamily	0.0087554	33.03	cl10023
159	48	115	YfbR superfamily	8.00E-06	43.91	cl43553
170	4	772	nrdA superfamily	0	844.00	cl33688
171	8	359	Ferritin_like superfamily	1.04E-98	297.26	cl00264
172	4	117	DUF3268 superfamily	9.69E-35	116.30	cl13172
173	3	67	Thioredoxin_like superfamily	1.11E-12	56.83	cl00388
180	3	421	30 superfamily	1.59E-102	313.18	cl33690
186	16	124	Nuc_deoxyri_tr2	8.17E-35	117.36	pfam15891
187	16	112	Nuc_deoxyri_tr2	1.24E-11	57.65	pfam15891
189	1	42	Zn-ribbon_8 superfamily	0.0006678	34.63	cl00993
191	1	150	RNaseH_like superfamily	9.37E-14	64.49	cl00861
193	1	30	cpn10 superfamily	0.0001025	37.02	cl09113
210	5	240	MPP_superfamily superfamily	8.82E-27	102.80	cl13995
218	11	156	MPP_superfamily superfamily	1.97E-24	92.81	cl13995
221	2	617	PTZ00108 superfamily	8.69E-179	543.49	cl36510
222	25	454	52 superfamily	6.84E-116	346.66	cl42980
230	1	288	pseT superfamily	1.11E-74	230.68	cl26099
233	15	326	DUF932 superfamily	4.10E-71	223.07	cl12129
235	25	211	Band_7	9.09E-14	67.35	pfam01145
238	15	132	PHA00684 superfamily	1.10E-40	131.90	cl10259
249	12	108	NTP-PPase_u2	3.88E-18	72.66	cd11539
254	21	171	ThyX superfamily	1.07E-06	48.20	cl42216
258	3	80	DUF4031	3.31E-25	89.22	pfam13223
261	18	145	TFold superfamily	9.56E-08	47.96	cl00263
266	1	96	Pyr_excise superfamily	3.08E-09	50.76	cl19573
269	2	384	PHA02142 superfamily	7.10E-58	192.85	cl40757
271	12	144	NADAR	8.76E-34	115.79	cd15457
280	197	519	COG5301 superfamily	4.47E-06	49.58	cl34977
281	1255	1317	Peptidase_S74	2.27E-07	48.78	pfam13884
286	1	206	COG3179 superfamily	6.89E-54	171.17	cl43803
288	84	197	Hydrolase_2	2.67E-33	114.54	pfam07486
290	26	318	RNA_lig_T4_1 superfamily	3.65E-40	145.97	cl09743
302	28	54	Ribosomal_L7_L12 superfamily	0.0013153	34.74	cl29562
305	1	288	cca superfamily	9.51E-111	328.74	cl42923
307	13	134	SegE_GIY-YIG	1.56E-42	137.02	pfam19835
309	63	191	19 superfamily	6.93E-07	47.35	cl28641
310	13	198	2 superfamily	4.08E-24	93.71	cl14353
311	7	145	Tn7_Tnp_TnsA_N superfamily	7.61E-53	164.06	cl21695

Supplementary Table S4. The tRNAs predicted in the phage X1 genome.

tRNA	start	stop	Function
1	123424	123496	tRNA-Val-GAC
2	123506	123580	tRNA-Leu-CAA
3	123809	123881	tRNA-Met-CAT
4	136052	136124	tRNA-Trp-CCA
5	136136	136208	tRNA-Cys-GCA
6	136222	136296	tRNA-Pro-TGG
7	136578	136650	tRNA-Ala-TGC
8	136665	136746	tRNA-Leu-GAG
9	137146	137218	tRNA-Thr-TGT
10	137230	137300	tRNA-Gly-TCC
11	137399	137470	tRNA-Glu-TTC
12	137480	137553	tRNA-Arg-TCT
13	137567	137649	tRNA-Tyr-GTA
14	137759	137831	tRNA-Val-TAC
15	138859	138933	tRNA-Glu-CTC
16	139177	139250	tRNA-Pro-TGG
17	139513	139595	tRNA-Asn-GTT
18	139605	139677	tRNA-Asn-GTT
19	139689	139760	tRNA-Asp-GTC
20	139944	140014	tRNA-Met-CAT
21	140107	140179	tRNA-Lys-CTT
22	140237	140318	tRNA-Lys-CTT
23	140850	140923	tRNA-Undet-NNN
24	141490	141572	tRNA-Leu-TAG
25	141583	141655	tRNA-Phe-GAA
26	141928	141999	tRNA-Phe-GAA
27	142276	142349	tRNA-Leu-CAG
28	142364	142437	tRNA-His-GTG
29	142487	142558	tRNA-Ile-GAT
30	142566	142636	tRNA-Ile-GAT
31	142839	142913	tRNA-Gln-TTG
32	142924	142994	tRNA-Gln-CTG
33	147122	147193	tRNA-Thr-GGT
34	147261	147334	tRNA-Gly-GCC

Supplementary Table S5. Prediction the auxiliary metabolic genes of phage X1.

KO Number	Gene Name	Annotated Function (EC Number)	Belonging Metabolic Pathway	Functional Category
K15789	TDH	threonine 3-dehydrogenase（EC:1.1.1.103）	Glycine, serine and threonine metabolism	Amino Acid Metabolism
K23370	serK	L-serine kinase (ADP)（EC:2.7.1.226）	Cysteine and methionine metabolism	Amino Acid Metabolism
K01940	argG	argininosuccinate synthase（EC:6.3.4.5）	Alanine, aspartate and glutamate metabolism	Amino Acid Synthesis
K01953	asnB	asparagine synthase (glutamine-hydrolyzing)（EC:6.3.5.4）	Alanine, aspartate and glutamate metabolism	Amino Acid Synthesis
K23977	GTK	L-glutamine:4-(methylthio)-2-oxobutanoate transaminase（EC:2.6.1.117）	Cysteine and methionine metabolism	Amino Acid Synthesis (Methionine Precursor)
K13657	gumH	alpha-1,3-mannosyltransferase（EC:2.4.1.252）	Exopolysaccharide biosynthesis	Exopolysaccharide Synthesis
K13684	wcaC	putative colanic acid biosynthesis glycosyltransferase WcaC（EC:2.4.-.-）	Exopolysaccharide biosynthesis	Exopolysaccharide Synthesis
K00953	FLAD1	FAD synthase（EC:2.7.7.2）	Riboflavin metabolism	Coenzyme Synthesis
K11753	ribF	riboflavin kinase/FMN adenylyltransferase（EC:2.7.1.26,2.7.7.2）	Riboflavin metabolism	Coenzyme Synthesis
K00763	pncB	nicotinate phosphoribosyltransferase（EC:6.3.4.21）	Nicotinate and nicotinamide metabolism	Coenzyme Synthesis (NAD/NADH)
K00952	nadM	nicotinamide nucleotide adenylyltransferase（EC:2.7.7.1）	Nicotinate and nicotinamide metabolism	Coenzyme Synthesis (NAD/NADH)
K00969	nadD	nicotinate nucleotide adenylyltransferase（EC:2.7.7.18）	Nicotinate and nicotinamide metabolism	Coenzyme Synthesis (NAD/NADH)
K01916	nadE	NAD+ synthase（EC:6.3.1.5）	Nicotinate and nicotinamide metabolism	Coenzyme Synthesis (NAD/NADH)
K03462	NAMPT	nicotinamide phosphoribosyltransferase（EC:2.4.2.12）	Nicotinate and nicotinamide metabolism	Coenzyme Synthesis (NAD/NADH)
K00859	coaE	dephospho-CoA kinase（EC:2.7.1.24）	Pantothenate and CoA biosynthesis	Coenzyme Synthesis (Coenzyme A)
K03148	thiF	sulfur carrier protein ThiS adenylyltransferase（EC:2.7.7.73）	Thiamine metabolism	Coenzyme Synthesis (Thiamine)
K05776	modF	molybdate transport system ATP-binding protein	ABC transporters	Ion Transport
K06857	tupC	tungstate transport system ATP-binding protein（EC:7.3.2.6）	ABC transporters	Ion Transport
K10824	nikE	nickel transport system ATP-binding protein（EC:7.2.2.11）	ABC transporters	Ion Transport
K15497	wtpC	molybdate/tungstate transport system ATP-binding protein（EC:7.3.2.5,7.3.2.6）	ABC transporters	Ion Transport
K00957	cysD	adenylylsulfate kinase subunit 2（EC:2.7.7.4）	Purine metabolism、Sulfur metabolism、Sulfur cycle	Sulfur Metabolism
K00390	cysH	adenosine 3',5'-bisphosphate sulfotransferase（EC:1.8.4.8,1.8.4.10）	Sulfur metabolism、Sulfur cycle	Sulfur Metabolism
K06074	btuD	vitamin B12 transport system ATP-binding protein（EC:7.6.2.8）	ABC transporters	Vitamin Transport
K16786	ecfA1	energy-coupling factor transport system ATP-binding protein（EC:7.-.-.-）	ABC transporters	Substance Transport
K03269	lpxH	UDP-2,3-diacylglucosamine hydrolase（EC:3.6.1.54）	Lipopolysaccharide biosynthesis	Cell Wall/Membrane Metabolism
K00712	tagE	poly(glycerol-phosphate) alpha-glucosyltransferase（EC:2.4.1.52）	Teichoic acid biosynthesis	Cell Wall Synthesis
K19002	mgs	1,2-diacylglycerol 3-alpha-glucosyltransferase（EC:2.4.1.337）	Teichoic acid biosynthesis	Cell Wall Synthesis
K22710	tarM	poly(ribitol-phosphate) alpha-N-acetylglucosaminyltransferase（EC:2.4.1.70）	Teichoic acid biosynthesis	Cell Wall Synthesis
